# Mechanistic Insights Into 5′‐tiRNA‐His‐GTG Mediated Activation of the JNK Pathway in Skin Photoaging

**DOI:** 10.1111/acel.70049

**Published:** 2025-03-17

**Authors:** Lihao Liu, Zhuohong Xu, Xiaoxi Dai, Xuyue Zhou, Lihao Chen, Chao Luan, Dan Huang, Hongying Chen, Jiaan Zhang, Yu Hu, Kun Chen

**Affiliations:** ^1^ Department of Physiotherapy, Hospital for Skin Diseases, Institute of Dermatology Chinese Academy of Medical Sciences & Peking Union Medical College Nanjing China

**Keywords:** JNK pathway, NUP98, photoaging, tiRNAs, tsRNAs

## Abstract

UV exposure leads to skin damage, thus inducing skin aging. The aims of this study were to explore the differences in tRNA‐derived small RNAs (tsRNAs) expression in the Human dermal fibroblasts (HDF cells) photoaging cell model and to explore the biological functions of tsRNA in skin photoaging. In this study, we found that in both photoaging cell models and the skin of photoaging mice, the 5′‐tiRNA‐His‐GTG expression levels were significantly elevated. In HDF cells, overexpression of 5′‐tiRNA‐His‐GTG induces cellular senescence. Inhibition of 5′‐tiRNA‐His‐GTG attenuates UVB‐induced cellular senescence in the photoaging cell model. Intradermal injection of Adeno‐associated virus 9‐5′‐tiRNA‐His‐GTG ‐Inhibition ameliorates UVB‐induced skin photoaging in nude mice. We confirmed that 5′‐tiRNA‐His‐GTG targeted nuclear pore proteins 98, which further activated the JNK signaling pathway and induced cell senescence. Targeting 5′‐tiRNA‐His‐GTG may provide a novel therapeutic option for ameliorating skin photoaging.

## Graphical abstract

5′‐tiRNA‐His‐GTG expression level is upregulated in the photoaging cell model. 5′‐tiRNA‐His‐GTG negatively regulates the expression of NUP98, thereby activating the JNK signaling pathway to regulate cell senescence.

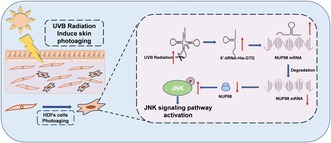

## 1 Introduction

Skin aging can be classified into two categories: chronological aging and extrinsic aging. Extrinsic skin aging is mainly associated with prolonged ultraviolet (UV) exposure and is known as skin photoaging. The manifestations of photodamaged skin include epidermal thickening, dryness, deepening of wrinkles, abnormal pigmentation, and increased risk of skin tumors (Soheilifar et al. 2022). Human dermal fibroblasts (HDF cells) are the primary cell type in the dermis and are responsible for synthesizing extracellular matrix (ECM). HDF cells are used in cellular senescence studies, and they exhibit a senescent‐like phenotype after UV radiation (Fitsiou et al. 2021).

Non‐coding RNAs (ncRNAs) are significant in the onset and progression of skin photoaging. Various ncRNAs are aberrantly expressed and regulate downstream target genes during skin photoaging, influencing cellular senescence, collagen synthesis, and several different signaling pathways (Soheilifar et al. 2022). tRNA‐derived small RNAs (tsRNAs) are a novel kind of ncRNAs derived from specific endonuclease processing of precursor or mature tRNAs, and based on the length of tsRNAs and the cleavage site of tRNA, tsRNAs can be categorized into tRNA fragments (tRFs) and tRNA‐derived stress‐induced small RNAs (tiRNAs). tRFs are 14–30 nt long tsRNAs derived from mature tRNA and contain the 5′ or 3′‐end region of the source tRNA. tiRNAs are a kind of 30–40 nt long ncRNA molecules produced by cleavage at the tRNA anticodon region (Lee et al. 2023).

Studies have demonstrated the involvement of tsRNAs in a variety of diseases, including tumors, cardiovascular diseases, and metabolic disorders (Zhang et al. 2023). In senescence‐related diseases, the expression levels of tsRNAs have been detected to have changed during aging and may be involved in regulating the cellular senescence process (Ha and Lee 2023).

The nuclear pore complex (NPC) is a multiprotein aqueous channel that connects the nucleoplasm to the cytoplasm. NPC is engaged in transporting material, regulating gene expression, and repairing DNA (Beck and Hurt 2017). NPC is composed of over 30 kinds of different nuclear pore proteins (NUP). It has been suggested that NPC may accumulate substantial biochemical damage and contribute to aging. NUP98 is one of the components of NPC, and previous studies have indicated that its expression level is reduced in senescent skin fibroblasts (Kim et al. 2010).

Currently, few studies have clarified the association or regulatory mechanism between tsRNAs and skin photoaging. In this study, we used UVB radiation to induce an HDF cell photoaging cell model. Using non‐coding RNA sequencing, we found that the tsRNAs expression profiles were markedly different between the photoaging cell model and the control group. Among them, 5′‐tiRNA‐His‐GTG expression was increased in the photoaging cell model. Overexpressing 5′‐tiRNA‐His‐GTG induced a senescent phenotype in HDF cells. 5′‐tiRNA‐His‐GTG targeted and reduced Nup98 expression levels through post‐transcriptional silencing, which in turn affected cellular senescence. Inhibition of 5′‐tiRNA‐His‐GTG could alleviate UVB‐induced skin photoaging in nude mice.

## 2 Results

### 2.1 Construction of UVB‐induced HDF photoaging cell model and animal model

In a previous study, it was found that a one‐time UVB radiation (30 mJ/cm^2^) to in vitro cultured HDF cells could establish the skin photoaging cell model (Chen et al. 2024). And 24 h after UVB radiation, the photoaging HDF model presented classic morphological alterations of senescent cells, including cell enlargement and flattening. Utilizing senescence‐associated β‐galactosidase (SA‐β‐gal) staining showed that the proportion of positive cells in the HDF photoaging model was higher than that of the control group (Figure S1A). The mRNA expression levels of the senescence‐associated secretory phenotype (SASP), such as *Interleukin* (*IL*)*‐1β*, *IL6*, and *IL8*, were significantly increased in the photoaging model (Figure S1B). The Western blotting (WB) assay showed that the classical senescence markers p53 and p21 were upregulated and the expression level of collagen type I was decreased in the photoaging model (Figure S1C). The cell proliferative capacity in the photoaging model was found to be significantly decreased using the EdU assay (Figure S1D).

Nude mice were used in order to establish the UVB‐induced skin photoaging animal model with the radiation protocol illustrated in the schematic diagram (Figure S1E). After receiving 5 weeks of UVB radiation, the dorsal skin of mice in the UVB‐radiated group exhibited deepening of skin wrinkles and significantly enhanced transepidermal water loss (TWEL) (Figure S1F,J). After acquiring dorsal skin specimens from both groups of nude mice, the samples were analyzed with H&E and Masson staining (Figure S1G). In the photoaging animal model group, the epidermis was thickened, and the collagen density in the skin was reduced (Figure S1H,I). To sum up, the photoaging HDF model and skin photoaging nude mouse model were successfully established using UVB radiation in HDF cells and nude mice.

### 2.2 tsRNAs Expressed Differently in the Photoaging HDF Cell Model, and tiRNAs Were Upregulated

**FIGURE 1:**
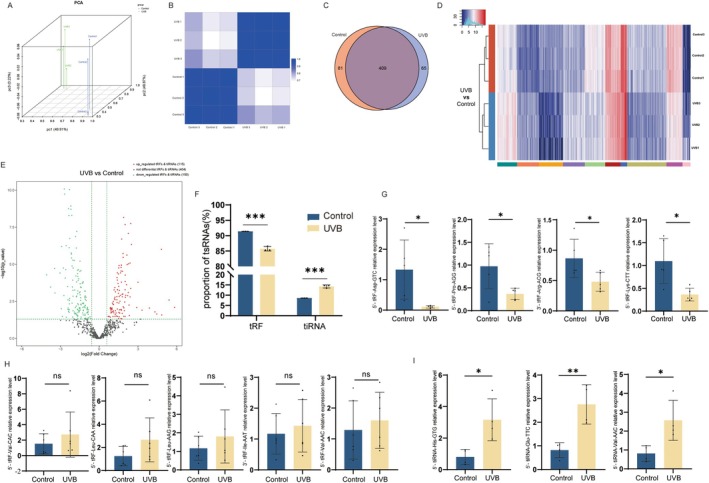
Differential expression Profile of tsRNA between photoaging cell model and control HDF cells samples. (A) PCA result. The X, Y, and Z axes represent the three main factors affecting the expression level of the sample. (B) Heatmap of the correlation coefficient for expression levels in the photoaging cell model and control HDF cell samples. (C) A Venn diagram showing tsRNAs on the basis of common and specific expressions between the two groups. (D) Heat map showing clustering of differentially expressed tsRNAs. (E) The volcano plot of differentially expressed tsRNAs. (F) The proportion of tRFs and tiRNAs between the photoaging model and the control group. (G) qRT‐PCR validation of downregulated tRFs from sequencing data. (H) qRT‐PCR validation of upregulated tRFs from sequencing data. (I) qRT‐PCR validation of upregulated tiRNAs from sequencing data.

tsRNAs have been demonstrated to engage in cellular senescence in previous studies (Ha and Lee 2023). In order to identify and characterize these differentially expressed tsRNAs, we obtained and compared the expression profiles of tsRNAs between the photoaging HDF cells model group and the control group with tsRNA sequencing. Through PCA analysis and correlation coefficient analysis, the correlation between the photoaging model group and the control group revealed that the expression profiles of tsRNAs in the two groups were significantly different (Figure 1A,B). Further analysis revealed that, as shown in the Venn diagram, there were 409 tsRNAs commonly expressed in both groups. 65 were specifically expressed only in the photoaging model group, while 81 were expressed only in the control group (Figure 1C). Hierarchical clustering heatmaps showed the overall expression profiles of tsRNAs in both groups (Figure 1D). In total, there were 265 differentially expressed tsRNAs. In comparison with the control group, the expression of 115 tsRNAs was up‐regulated and 150 tsRNAs were down‐regulated in the photoaging HDF cells model group (Figure 1E).

We further analyzed the expression profiles of tsRNAs of both groups. Due to the fact that tiRNA is associated with various factors such as hypoxia and stress, we compared the percentage of tRFs versus tiRNAs in tsRNAs in both groups, and the percentage of tiRNA in tsRNAs was markedly increased in the photoaging model group (Figure 1F). Next, we performed qRT‐PCR on the tRFs with the top‐ranked expression differences and found that the expression of 5′‐tRF‐Asp‐GTC, 5′‐tRF‐Pro‐AGG, 3′‐tRF‐Arg‐ACG, and 5′‐tRFRF‐Lys‐CTT was markedly reduced in the photoaging cell model (Figure 1G). Meanwhile, the expression of 5′‐tRF‐Val‐CAC, 5′‐tRF‐Leu‐CAA, 5′‐tRF‐Leu‐AAG, 3′‐tRF‐Ile‐AAT, and 5′‐tRF‐Val‐AAC was increased in the photoaging cell model (Figure 1H). Also, the expression of 5′‐tiRNA‐His‐GTG, 5′‐tiRNA‐Glu‐TTC, and 5′‐tiRNA‐Val‐AAC was also elevated in the photoaging cell model (Figure 1I). This evidence illustrates clearly that the tsRNAs expression of the photoaging cell model group and the control group is markedly different, and tiRNAs exerted an important role in the photoaging cell model.

### 2.3 Overexpressing 5′‐tiRNA‐His‐GTG in HDF Cells Induced Cellular Senescence

**FIGURE 2:**
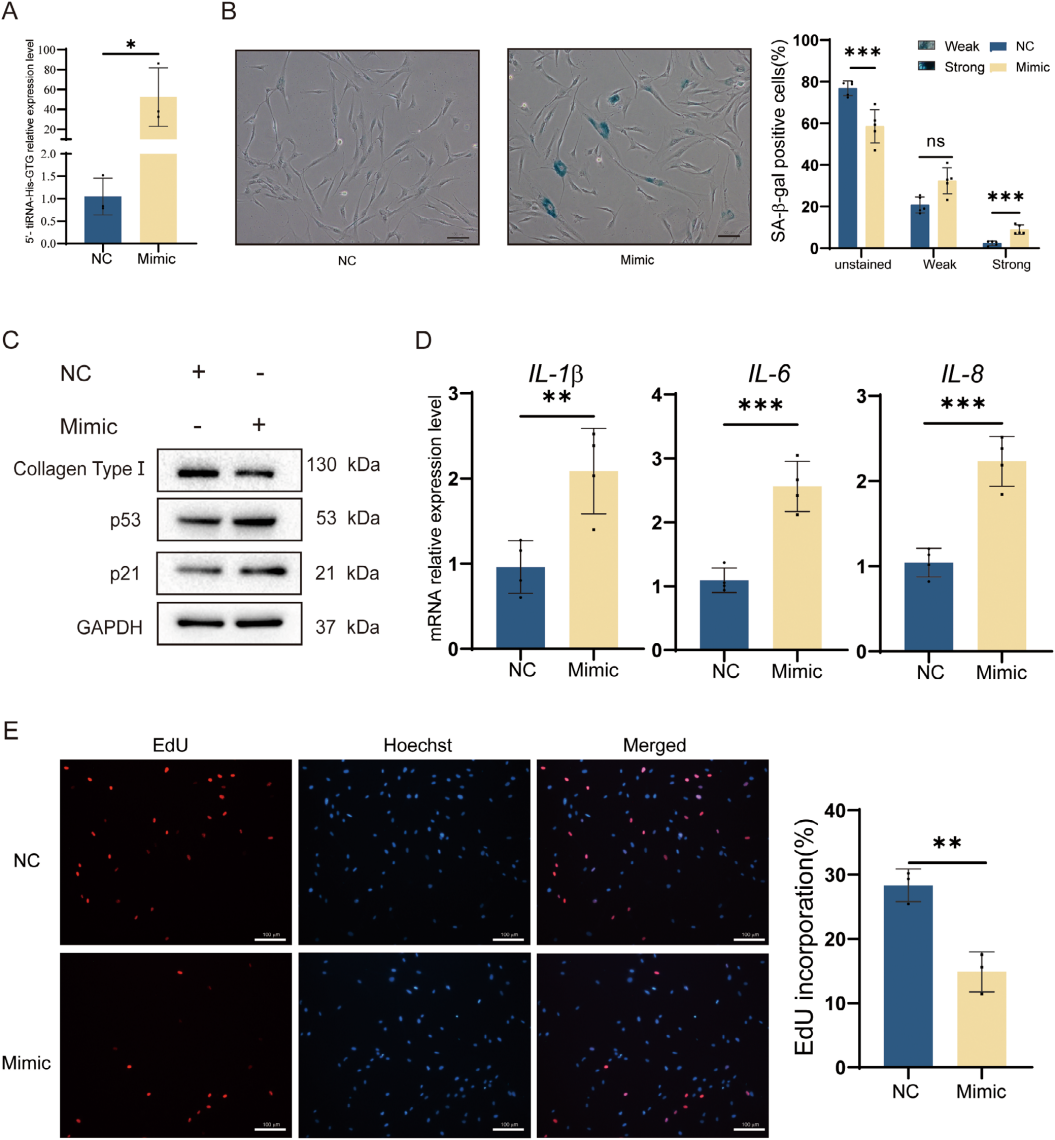
Overexpression of 5′‐tiRNA‐His‐GTG in HDF cells induced cellular senescence. (A) qRT‐PCR validation of the overexpression of 5′‐tiRNA‐His‐GTG in HDF cells. (B) Overexpressing 5′‐tiRNA‐His‐GTG in HDF cells and staining with SA‐β‐gal after 24 h. Cells with blue staining represent senescent cells. The treated cells were divided into three groups: unstained, strongly positive, and weakly positive. The percentage of cells in each group was presented in a bar graph. The scale bar, 100 μm. (C) WB analysis of Collagen Type I, p53, and p21 in HDF cells after 5′‐tiRNA‐His‐GTG Mimic transfection. (D) The mRNA levels of *IL*‐*1β*, *IL*‐*6*, and *IL*‐*8* in HDF cells after 5′‐tiRNA‐His‐GTG Mimic transfection. (E) EdU assay was used to analyze HDF cells' proliferation ability after overexpressing 5′‐tiRNA‐His‐GTG. The scale bar is 100 μm.

To explore tiRNAs' function in photoaging cell models, we chose the top differentially expressed 5′‐tiRNA‐His‐GTG as a target tiRNA for further studies. The 5′‐tiRNA‐His‐GTG was overexpressed in HDF cells (Figure 2A) and cellular senescence was observed. SA‐β‐gal staining assay revealed that the proportion of positive senescent cells increased with overexpressing 5′‐tiRNA‐His‐GTG (Figure 2B). Overexpression of 5′‐tiRNA‐His‐GTG in HDF cells induced p53 and p21 expression and reduction of collagen type I (Figure 2C). It was found that overexpression of 5′‐tiRNA‐His‐GTG in HDF cells upregulated *IL‐1β*, *IL‐6*, and *IL‐8* mRNA expression levels (Figure 2D). EdU assay revealed that overexpression of 5′‐tiRNA‐His‐GTG inhibited the proliferation of HDF cells, and the percentage of EdU positive cells was significantly reduced (Figure 2E). In summary, 5′‐tiRNA‐His‐GTG induced senescence in HDF cells.

### 2.4 Inhibition of 5′‐tiRNA‐His‐GTG Rescued UVB‐Induced HDF Cell Photoaging

**FIGURE 3:**
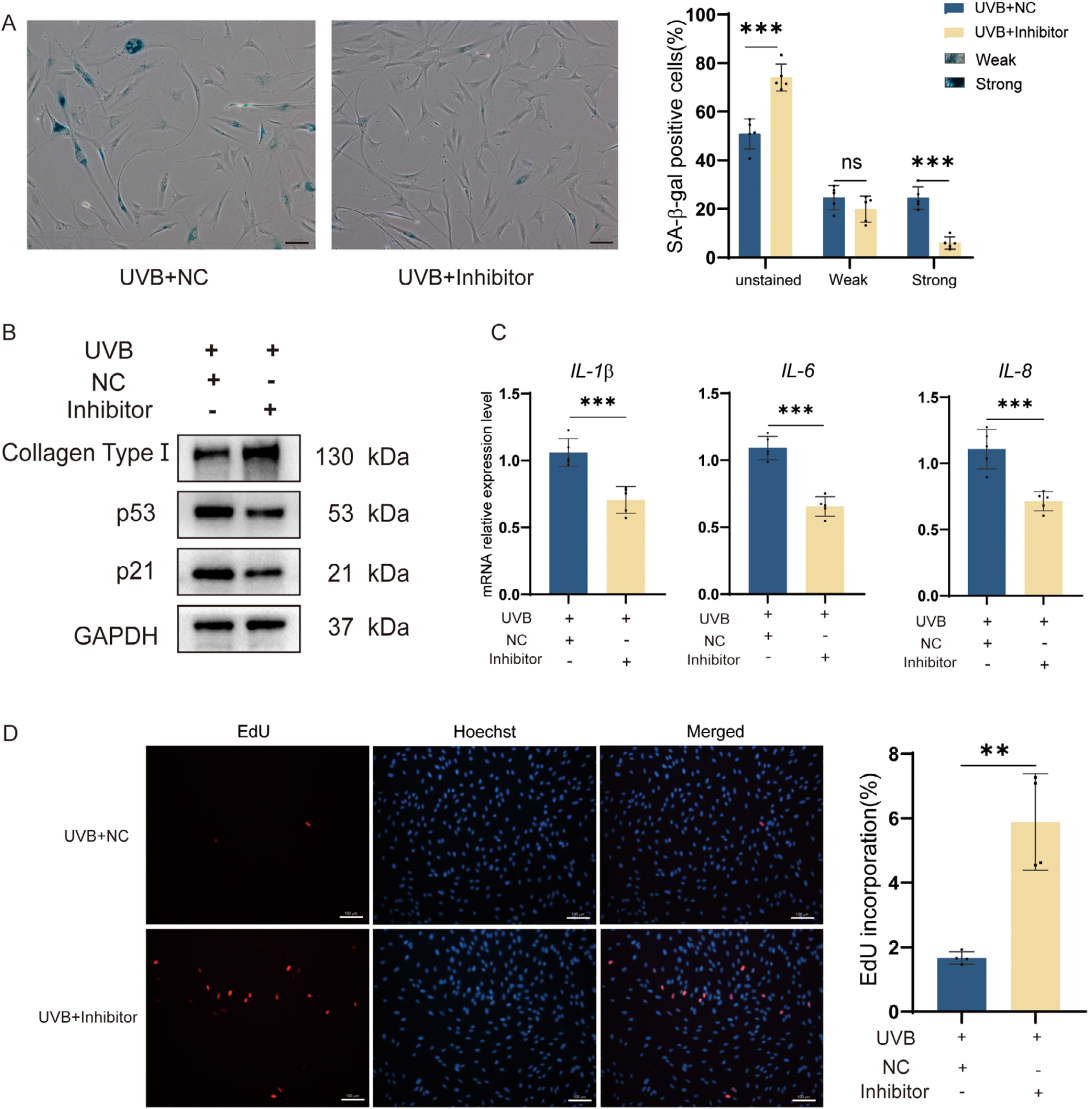
Inhibiting 5′‐tiRNA‐His‐GTG rescued UVB‐induced HDF cells photoaging. (A) HDF cells were exposed to UVB after inhibiting 5′‐tiRNA‐His‐GTG, and stained with SA‐β‐gal after 24 h. Cells with blue staining represent senescent cells. The treated cells were divided into three groups: unstained, strongly positive, and weakly positive. The percentage of cells in each group was presented in a bar graph. The scale bar, 100 μm. (B) WB analysis of Collagen Type I, p53, and p21 in the photoaging cell model after 5′‐tiRNA‐His‐GTG Inhibitor transfection. (C) The mRNA levels of *IL*‐*1β*, *IL*‐*6*, and *IL*‐*8* in the photoaging cell model after 5′‐tiRNA‐His‐GTG Inhibitor transfection. (D) EdU assay was used to analyze cell proliferation ability in the photoaging cell model after 5′‐tiRNA‐His‐GTG Inhibitor transfection. The scale bar, 100 μm.

The expression level of 5′‐tiRNA‐His‐GTG was elevated in the HDF photoaging model, and overexpression of 5′‐tiRNA‐His‐GTG induced a senescent phenotype in HDF cells. Subsequently, we treated the HDF photoaging cell model with the 5′‐tiRNA‐His‐GTG Inhibitor and monitored changes in the photoaging phenotype. The expression levels of source tRNA‐His‐GTG were not affected by the 5′‐tiRNA‐His‐GTG Inhibitor (Figure S2). The percentage of UVB‐induced senescent cells was reduced with the use of the 5′‐tiRNA‐His‐GTG Inhibitor, as proven by SA‐β‐gal staining (Figure 3A). Inhibition of 5′‐tiRNA‐His‐GTG suppressed the up‐regulation of p21 and p53 expression levels in addition to rescuing the UVB‐induced decrease in collagen type I levels (Figure 3B). Similarly, the use of the 5′‐tiRNA‐His‐GTG Inhibitor suppressed the mRNA levels of *IL‐1β*, *IL‐6*, and *IL‐8* compared with the Inhibitor NC group (Figure 3C). EdU assay indicated that using the 5′‐tiRNA‐His‐GTG Inhibitor upregulated the decreased proliferative ability of the photoaging cell model (Figure 3D). Collectively, the use of the 5′‐tiRNA‐His‐GTG Inhibitor was effective in rescuing the UVB‐induced HDF cells photoaging.

### 2.5 5′‐tiRNA‐His‐GTG Directly Targeted and Regulated NUP98 Expression in HDF Cells

**FIGURE 4:**
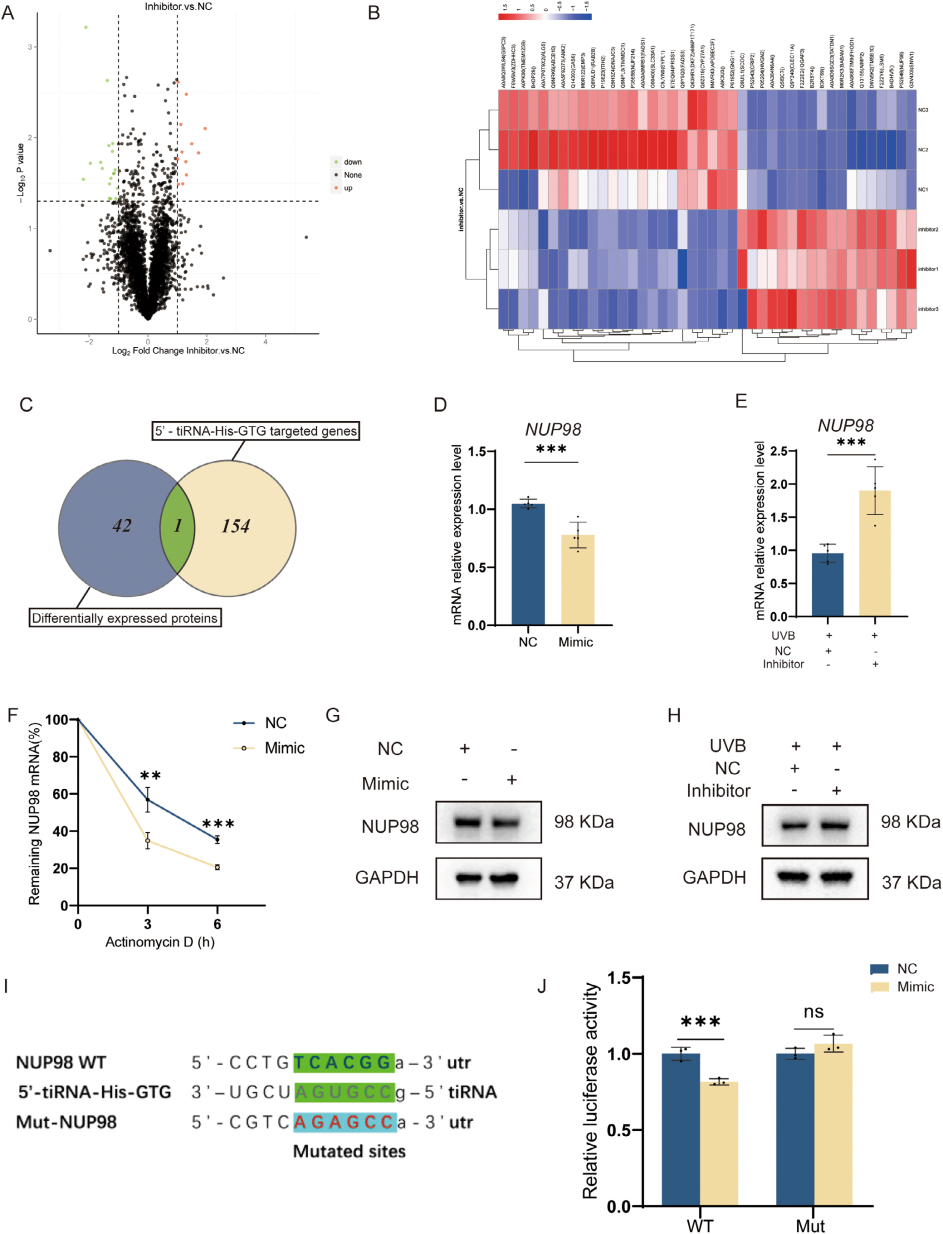
5′‐tiRNA‐His‐GTG directly regulated NUP98 expression in HDF cells. (A) A Volcano plot of differentially expressed proteins in the HDF photoaging cell model treated with 5′‐tiRNA‐His‐GTG Inhibitor or Inhibitor NC. (B) A hierarchical clustering heatmap of differentially expressed proteins. (C) A Venn diagram showing shared target genes between differentially expressed proteins in the photoaging cell model treated with 5′‐tiRNA‐His‐GTG Inhibitor and 5′‐tiRNA‐His‐GTG target gene prediction. (D) The mRNA levels of *NUP98* in HDF cells after being transfected with 5′‐tiRNA‐His‐GTG mimic. (E) The mRNA levels of *NUP98* mRNA in the photoaging cell model treated with 5′‐tiRNA‐His‐GTG Inhibitor. (F) HDF cells transfected with 5′‐tiRNA‐His‐GTG mimic or NC were treated with actinomycin D (10 μg/mL) for 0, 3, and 6 h, and *NUP98* mRNA stability was assessed. (G) WB analysis of NUP98 in HDF cells after being transfected with 5′‐tiRNA‐His‐GTG mimic. (H) WB analysis of NUP98 in the photoaging cell model treated with 5′‐tiRNA‐His‐GTG Inhibitor. (I) The predicted binding sequence in the 3′‐UTR of NUP98 for 5′‐tiRNA‐His‐GTG and its mutant sequence contained in luciferase vectors. (J) Dual‐luciferase reporter assay shows 5′‐tiRNA‐His‐GTG mediated inhibition of the activity of wild‐type NUP98 mRNA. wildtype, WT. mutant, MUT.

By using the Label‐free proteomics technique, we further investigated the role of 5′‐tiRNA‐His‐GTG in photoaging. Protein expression analysis was performed on HDF photoaging cell models treated with 5′‐tiRNA‐His‐GTG Inhibitor or Inhibitor NC. Compared with using the Inhibitor NC, 42 proteins expressed differently after treatment with the 5′‐tiRNA‐His‐GTG inhibitor in the photoaging cell model. Around 18 of the differentially expressed proteins (DEPs) were up‐regulated and 24 DEPs were down‐regulated (Figure 4A,B).

We performed further analysis of differential protein expression in HDF photoaging cell models treated with 5′‐tiRNA‐His‐GTG Inhibitor or Inhibitor NC. A total of 154 genes were predicted as the 5′‐tiRNA‐His‐GTG target genes using miRanda and TargetScan databases. We found one target gene that was also listed among the differentially expressed proteins, which is nuclear pore protein 98 (NUP98) (Figure 4C). In HDF cells overexpressing 5′‐tiRNA‐His‐GTG, *NUP98* mRNA, and NUP98 protein levels were decreased (Figure 4D,G). The *NUP98* mRNA and NUP98 protein levels were elevated in the HDF photoaging cell model using the 5′‐tiRNA‐His‐GTG Inhibitor in comparison to using inhibitorNC (Figure 4E,H). After transfection of 5′‐tiRNA‐His‐GTG mimic or NC into HDF cells, cells were treated with actinomycin D to stop transcription. 5′‐tiRNA‐His‐GTG mimic destabilized *NUP98* mRNA (Figure 4F). In this study, NUP98 was chosen as the gene of interest for further study. In HEK293T cells, the dual luciferase reporter gene assay was performed and it demonstrated that 5′‐tiRNA‐His‐GTG binds to *NUP98* mRNA 3′‐untranslated region (3′‐UTR) and inhibits its translational process, resulting in a decrease in fluorescence intensity (Figure 4I,J). Therefore, NUP98 has been identified as the target mRNA of 5′‐tiRNA‐His‐GTG to participate in its regulation of cell senescence.

### 2.6 Overexpression of NUP98 Rescues 5′‐tiRNA‐His‐GTG‐Induced Cellular Senescence in HDF Cells

**FIGURE 5:**
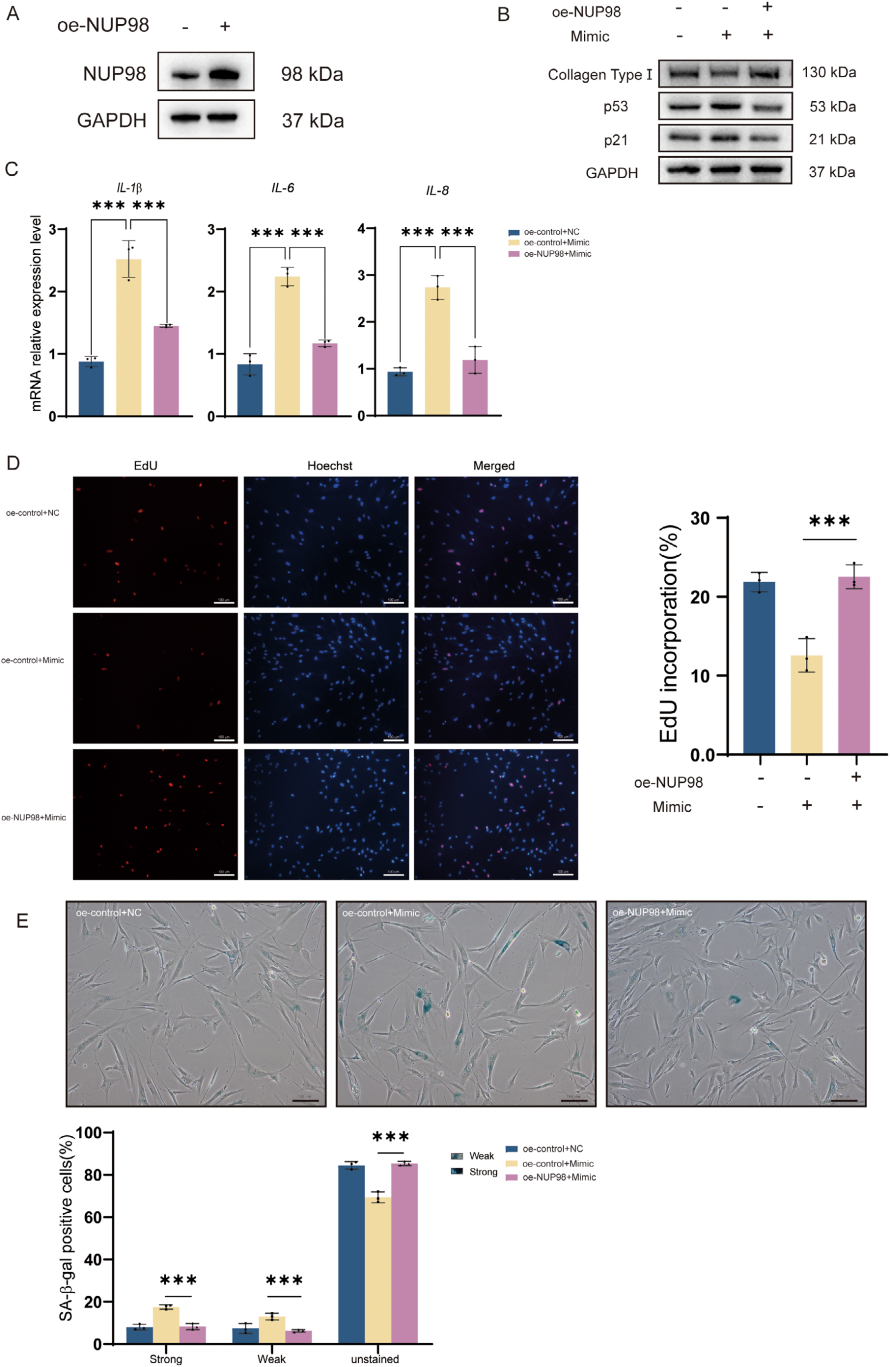
Overexpression of NUP98 rescues 5′‐tiRNA‐His‐GTG‐induced cell senescence in HDF cells. (A) WB analysis of oe‐NUP98 in HDF cells. (B) WB analysis of Collagen Type I, p53 and p21 in HDF cells and oe‐NUP98 HDF cells, with 5′‐tiRNA‐His‐GTG Mimic transfection. (C) The mRNA levels of *IL*‐*1β*, *IL*‐*6* and *IL*‐*8* in HDF cells and oe‐NUP98 HDF cells, after 5′‐tiRNA‐His‐GTG Mimic transfection. (D) EdU assay was used to analyze HDF cells and oe‐NUP98 HDF cells proliferation ability after 5′‐tiRNA‐His‐GTG Mimic transfection. The scale bar, 100 μm. (E) HDF cells and oe‐NUP98 HDF cells were transfected with 5′‐tiRNA‐His‐GTG Mimic and stained with SA‐β‐gal after 48 h. Cells with blue staining represent senescent cells. The treated cells were divided into three groups: unstained, strongly positive, and weakly positive. The percentage of cells in each group was presented in a bar graph. The scale bar, 100 μm.

Since we demonstrated that 5′‐tiRNA‐His‐GTG directly targets the expression of NUP98, lentivirus was used to overexpress NUP98 (oe‐NUP98) in HDF cells (Figure 5A). Overexpression of NUP98 down‐regulated the rise in p53 and p21 caused by the 5′‐tiRNA‐His‐GTG mimic, as well as up‐regulated the expression of collagen type I compared to controls (Figure 5B). The overexpression of NUP98 reduced the upregulation of *IL‐1β*, *IL‐6*, and *IL‐8* mRNAs by the 5′‐tiRNA‐His‐GTG mimic (Figure 5C). The decrease in cell proliferation capacity caused by the 5′‐tiRNA‐His‐GTG mimic was recovered by the overexpression of NUP98(Figure 5D). As confirmed by SA‐β‐gal staining, the overexpression of NUP98 reduced the proportion of senescent cells caused by the 5′‐tiRNA‐His‐GTG mimic (Figure 5E). These results suggest that overexpressing NUP98 rescues 5′‐tiRNA‐His‐GTG‐induced cellular senescence in HDF cells.

### 2.7 Overexpression of NUP98 Attenuates Cellular Senescence

**FIGURE 6:**
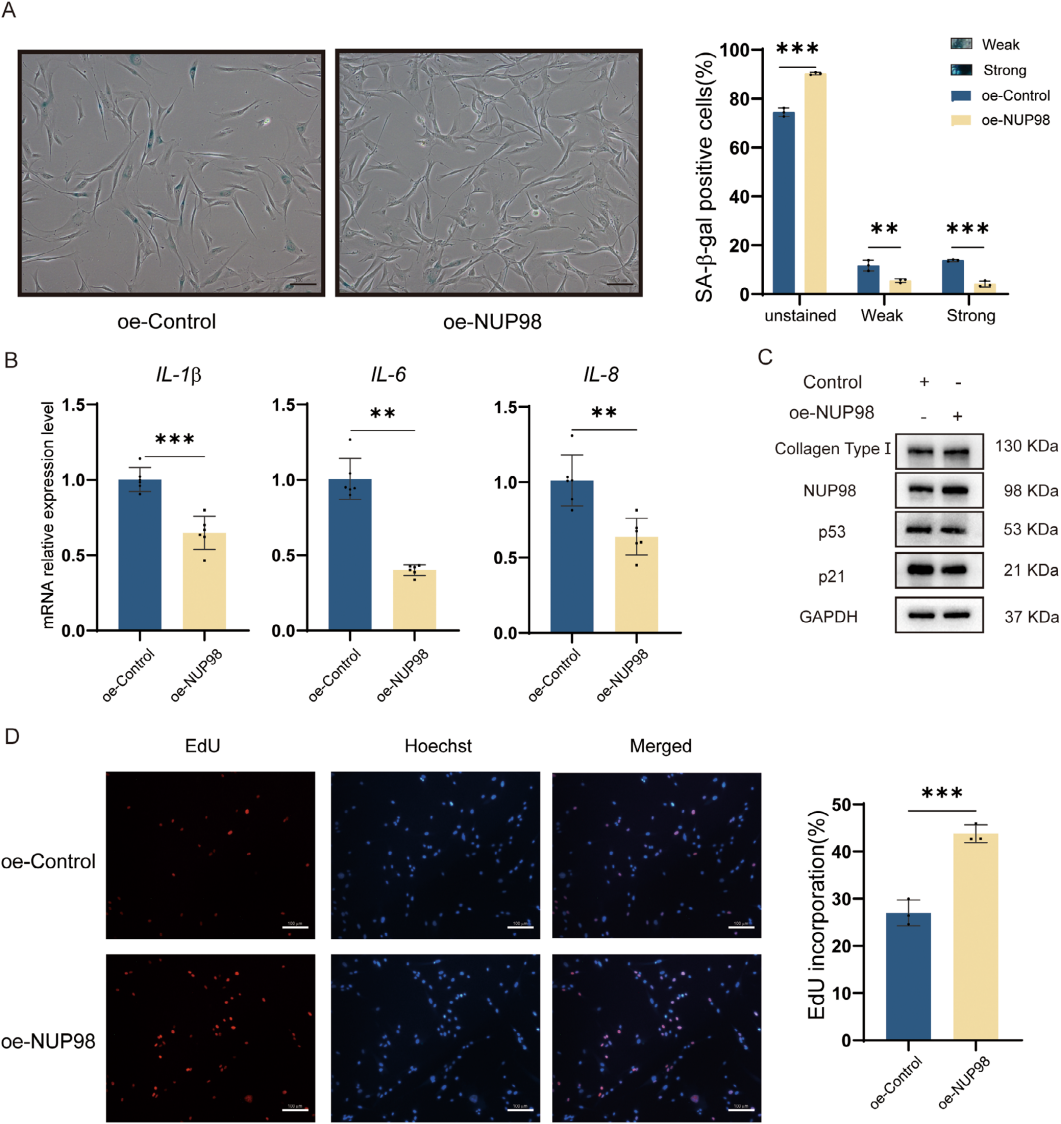
Overexpression of NUP98 attenuates cellular senescence. (A) oe‐NUP98 HDF cells were stained with SA‐β‐gal after 48 h. Cells with blue staining represent senescent cells. The treated cells were divided into three groups: unstained, strongly positive, and weakly positive. The percentage of cells in each group was presented in a bar graph. The scale bar is 100 μm. (B) The mRNA levels of *IL*‐*1β*, *IL*‐*6* and *IL*‐*8* in oe‐NUP98 HDF cells. (C) WB analysis of NUP98, Collagen Type I, p53 and p21 in oe‐NUP98 HDF cells. (D) EdU assay was used to analyze oe‐NUP98 HDF cells' proliferation ability. The scale bar is 100 μm.

To investigate the effect of NUP98 on HDF cells. Accordingly, we overexpressed NUP98 (Figure 5A) in HDF cells to observe its effect on cellular senescence. SA‐β‐gal staining showed that the proportion of senescent cells was lower in the NUP98 overexpression group (Figure 6A). The qRT‐PCR results revealed that the mRNA levels of *IL1β*, *IL‐6*, and *IL8* were reduced in the oe‐NUP98 HDF cells (Figure 6B). Also, overexpression of NUP98 up‐regulated the level of collagen type I while decreasing p53 and p21 (Figure 6C). Overexpression of NUP98 was shown to promote the proliferative capacity of HDF cells (Figure 6D). NUP98 overexpression also mitigated UVB‐induced cellular senescence (Figure S3). These results suggest that overexpression of NUP98 ameliorates cellular senescence, indicating that it is strongly involved in cellular senescence.

### 2.8 5′‐tiRNA‐His‐GTG/NUP98 Functions by Activating the JNK Signaling Pathway

**FIGURE 7:**
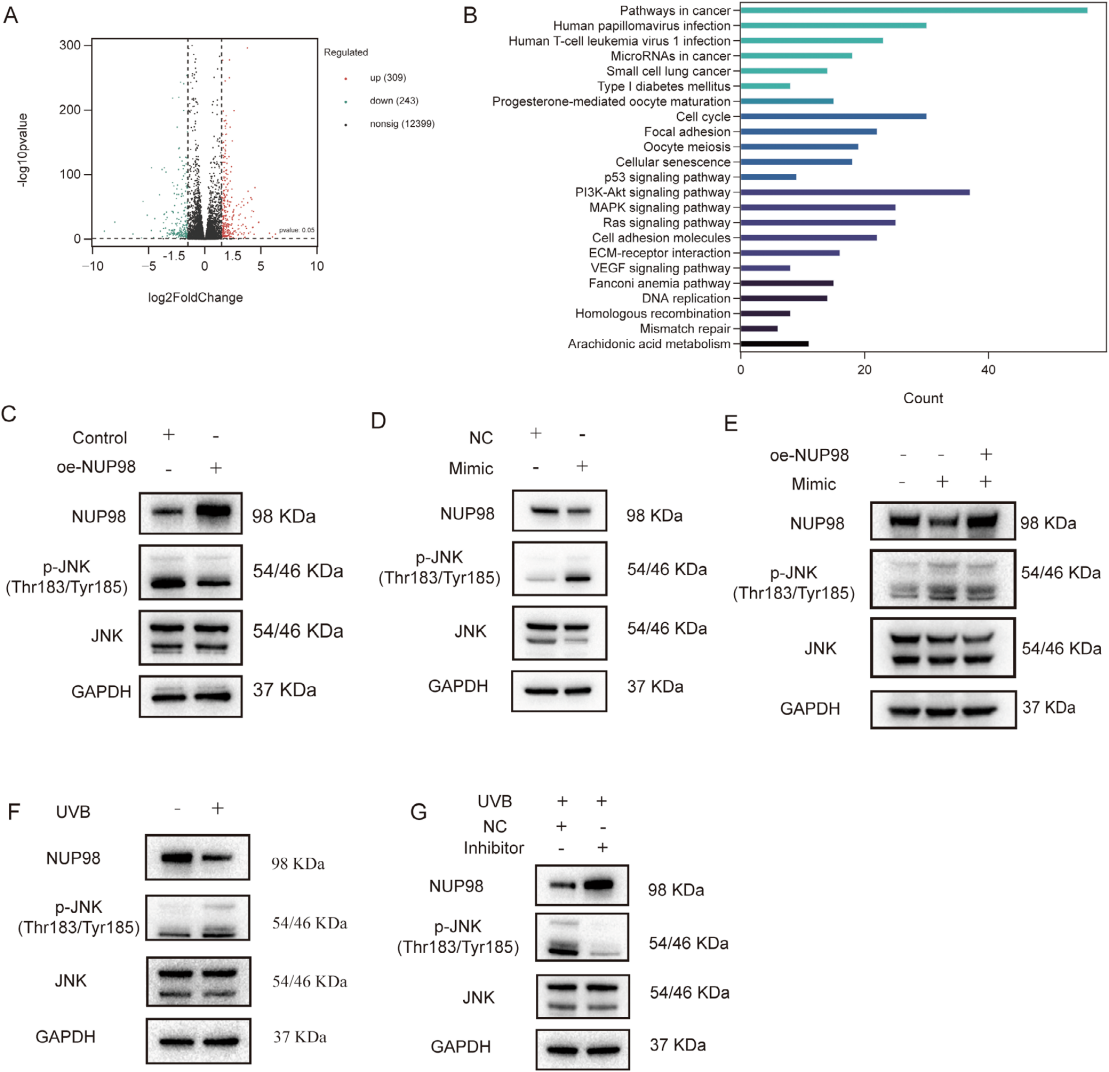
5′‐tiRNA‐His‐GTG/NUP98 functions by activating the JNK signaling pathway. (A) The volcano plot of differentially expressed RNA between oe‐NUP98 HDF cells and the control group. (B) KEGG enrichment analysis of DEGs in oe‐NUP98 HDF cells compared to the control group. (C) WB analysis of JNK signaling pathway activation in oe‐NUP98 HDF cells. (D) WB analysis of JNK signaling pathway activation in HDF cells after 5′‐tiRNA‐His‐GTG Mimic transfection. (E) WB analysis of JNK signaling pathway activation in HDF cells and oe‐NUP98 HDF cells, after 5′‐tiRNA‐His‐GTG Mimic transfection. (F) WB analysis of JNK signaling pathway activation in HDF cells after UVB radiation. (G) WB analysis of JNK signaling pathway activation in the photoaging cell model after 5′‐tiRNA‐His‐GTG Inhibitor transfection.

To further explore the mechanism underlying the amelioration of cellular senescence by overexpressing NUP98, we conducted high‐throughput mRNA sequencing of HDF cells overexpressing NUP98. A total of 309 genes were upregulated in expression and 243 genes were downregulated compared to the control group (Figure 7A). KEGG enrichment analysis revealed that these DEGs were enriched in cellular processes or signaling pathways associated with cellular senescence or photoaging, including cellular senescence, cell cycle, DNA replication, p53 signaling pathway, The phosphatidylinositol 3‐kinase (PI3K)/protein kinase B (AKT) signaling pathway, and mitogen‐activated protein kinase (MAPK) signaling pathway (Figure 7B). Studies have stated that the MAPK pathway plays a significant role in photoaging, and it has also been found that the knockdown of NUP98 in PC3 prostate cancer cells upregulates the level of c‐Jun N‐terminal kinase (JNK) phosphorylation and activates the MAPK pathway. The level of JNK phosphorylation was reduced in HDF cells overexpressing NUP98 (Figure 7C), suggesting inhibition of the JNK‐MAPK signaling pathway. The JNK signaling pathway was found to be activated in HDF cells after overexpression of 5′‐tiRNA‐His‐GTG, and the JNK phosphorylation level was downregulated in oe‐NUP98 HDF cell (Figure 7D,E). In the photoaging cell model group, the JNK signaling pathway was also activated, and the JNK signaling pathway was suppressed after 5′‐tiRNA‐His‐GTG Inhibitor transfection (Figure 7F,G). Furthermore, the use of a JNK pathway inhibitor can inhibit both 5′‐tiRNA‐His‐GTG‐induced activation of the JNK signaling pathway and 5′‐tiRNA‐His‐GTG mimic‐induced cellular senescence (Figure S4).

To investigate how NUP98 downregulates JNK phosphorylation, we performed quantitative ROS detection using Dihydroethidium fluorescence probes in oe‐NUP98 HDF cells. NUP98 overexpression significantly attenuated ROS accumulation compared to the control group (Figure S5). In summary, the above data indicate that UVB induces the 5′‐tiRNA‐His‐GTG elevation in HDF cells, which negatively regulates the expression of NUP98, and NUP98‐mediated ROS reduction attenuates JNK activation, thereby disrupting the ROS‐JNK‐senescence signaling axis.

### 2.9 Inhibition of 5′‐tiRNA‐His‐GTG Mitigates UVB‐Induced Skin Photoaging in Nude Mice

**FIGURE 8:**
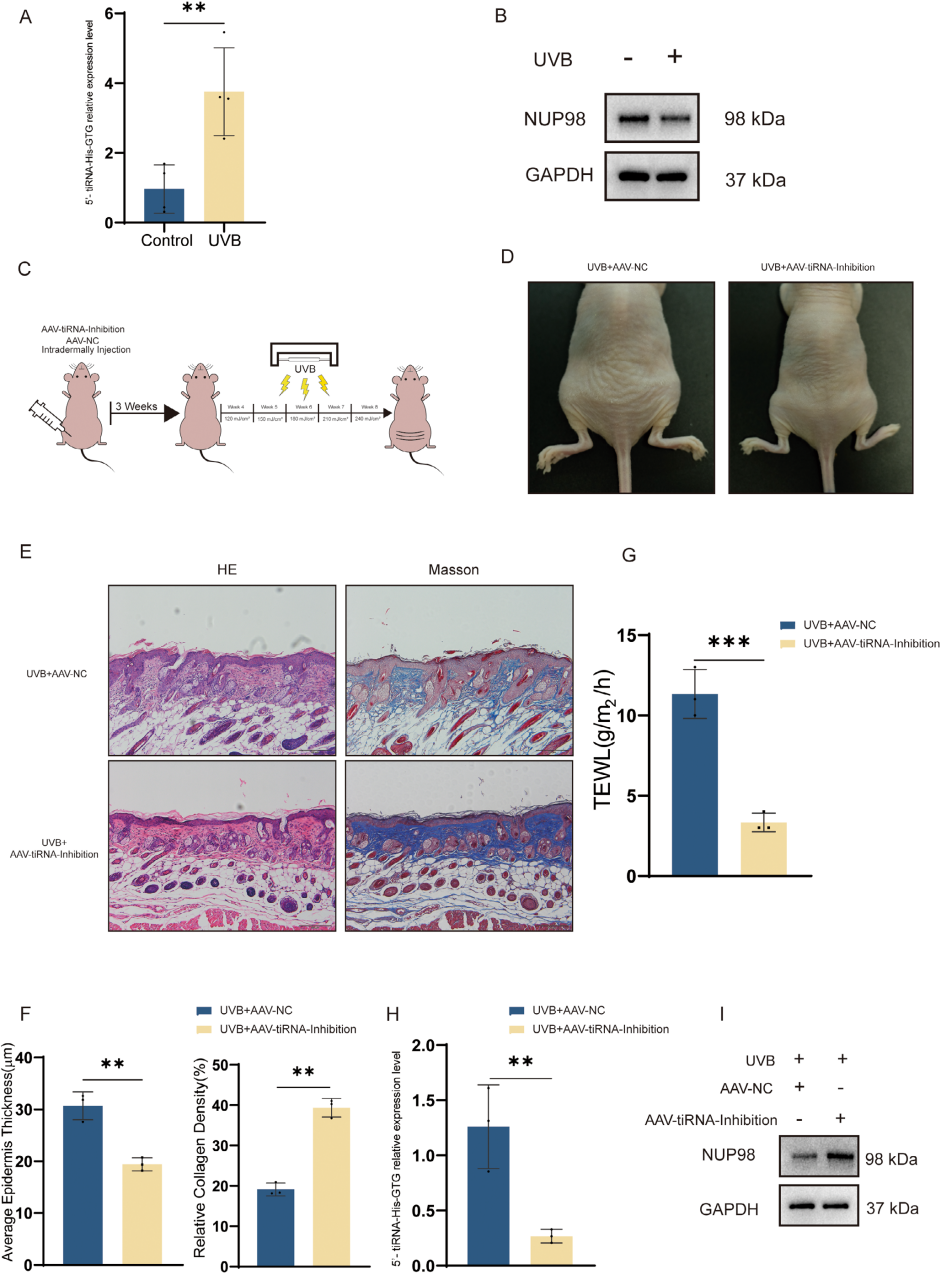
Inhibition of 5′‐tiRNA‐His‐GTG mitigates UVB‐induced skin photoaging in nude mice. (A) The expression levels of 5′‐tiRNA‐His‐GTG in the skin tissue of the control group and photoaging nude mice group (*n* = 4). (B) WB analysis of NUP98 in the skin tissue of the control group and photoaging nude mice group. (C) The Schematic diagram illustrated the procedure for indicated treatment before UVB radiation. (D) Representative images of dorsal skin of nude mice treated with AAV9‐tiRNA‐Inhibition after UVB radiation. (E) Representative section images of dorsal skin of nude mice treated with AAV9‐tiRNA‐Inhibition after UVB radiation, with H&E staining and Masson trichrome staining. (F) Epidermal thickness was measured in skin sections. Collagen density in mice skin sections was measured by ImageJ. (G) The degree of transdermal water loss in the dorsal skin of nude mice treated with AAV9‐tiRNA‐Inhibition after UVB radiation was measured using the gpskin machine. (H) The expression levels of 5′‐tiRNA‐His‐GTG in the skin tissue of nude mice treated with AAV9‐tiRNA‐Inhibition after UVB radiation and the control group (*n* = 3). (I) WB analysis of NUP98 in the skin tissue of nude mice treated with AAV9‐tiRNA‐Inhibition after UVB radiation and the control group. AAV, Adeno‐associated virus; AAV‐5′‐tiRNA‐His‐GTG‐inhibition; AAV9‐tiRNA‐inhibition.

Based on the previous findings, we performed qRT‐PCR validation in skin tissues of photoaging nude mice and found elevated expression of 5′‐tiRNA‐His‐GTG (Figure 8A), as well as decreased expression of NUP98 (Figure 8B), which was similar to the results of the photoaging HDF cell model. To further explore the effect of 5′‐tiRNA‐His‐GTG in photoaging, we injected AAV‐5′‐tiRNA‐His‐GTG‐Inhibition (AAV9‐tiRNA‐Inhibition) or AAV9‐NC intradermally into the dorsal skin of nude mice before proceeding to UVB radiation to induce skin photoaging as indicated (Figure 8C). EGFP fluorescence was detected in the dermis of the treated mice (Figure S6). Nude mice injected with AAV9‐tiRNA‐Inhibition had fewer skin wrinkles compared to those with AAV9‐NC (Figure 8D). The epidermal thickness of mice in the AAV9‐tiRNA‐Inhibition group was thinner than that in the AAV9‐NC group. Masson staining revealed a higher proportion of collagen fibers in the dermis of mice in the AAV9‐tiRNA‐Inhibition group (Figure 8E,F). Treatment with AAV9‐tiRNA‐Inhibition alleviates the level of transepidermal water loss in the dorsal skin of photodamaged nude mice (Figure 8G). Meanwhile, skin tissues were collected from both groups; the skin of mice in the AAV9‐tiRNA‐Inhibition group had a reduced level of expression of tiRNA‐His‐GTG and an elevated expression protein level of NUP98 (Figure 8H,I). These results revealed that UVB‐induced skin photoaging could be ameliorated by inhibiting 5′‐tiRNA‐His‐GTG.

## 3 Discussion

Overexposure to UV radiation is one of the major causes of premature skin aging (Ke and Wang 2021). HDF cells develop a cellular senescence phenotype after UV radiation, including elevated levels of p21 and p53, decreased levels of type I collagen, reduced cellular proliferative capacity, higher levels of SASP expression, and increased levels of SA‐β‐gal (Fitsiou et al. 2021). Recent studies on the mechanisms of skin photoaging are mainly focused on inflammation‐related pathways, oxidative stress, DNA damage and repair, extracellular matrix degradation, and non‐coding RNAs (Hajialiasgary et al. 2024; Russell‐Goldman and Murphy 2020).

While tsRNAs were initially considered to belong to randomly degraded fragments of tRNAs, studies have revealed that tsRNAs are important in various biological processes, including gene expression, apoptosis, and intergenerational inheritance (Kim et al. 2020). tiRNAs were found under different stress conditions, such as amino acid or glucose starvation, heat shock, hypoxia, and ultraviolet radiation. tRNAs can be specifically cleaved to form tiRNAs in the presence of specific enzymes, including angiopoietin (ANG), RNase T2, and RNase L (Kim et al. 2020). We used non‐coding RNA sequencing techniques to identify differentially expressed tsRNAs in the UVB‐induced HDF photoaging cell model, to search for potential markers of senescence and to investigate the mechanisms of UV‐induced skin aging (Figure 1). The proportion of tiRNA was found to be significantly elevated in the UVB‐induced HDF photoaging cell model (Figure 1F), and 5′‐tiRNA‐His‐GTG was upregulated in both the photoaging cell model (Figure 1I) and the animal model (Figure 8A).

Several studies have suggested that tsRNAs may serve as potential biological markers of aging. tiRNA can inhibit apoptosis by binding to cytochrome C, which may lead to cellular senescence (Saikia et al. 2014). In the brain, ANG mediates tRNA^Glu^ cleavage, leading to increased levels of age‐induced Glu‐5′tsRNA‐CTC. Glu‐5′tsRNA‐CTC inhibits mitochondrial‐tRNA^Leu^ binding to leucyl‐tRNA synthetase2, disrupting mitochondrial cristae organization, suppressing protein synthesis, and contributing to brain aging (Li et al. 2024). In this study, 5′‐tiRNA‐His‐GTG mimic was synthesized to increase its abundance in cells (Figure 2A). In vitro experiments revealed that 5′‐tiRNA‐His‐GTG induced a cellular senescence phenotype in HDF cells (Figure 2B–E). Inhibiting 5′‐tiRNA‐His‐GTG rescued the UVB‐induced senescence phenotype in the photoaging cell model (Figure 3A–D). Similarly, in vivo experiments, inhibition of 5′‐tiRNA‐His‐GTG using AAV ameliorated UVB‐induced skin aging in nude mice (Figure 8D–G). These results prove that 5′‐tiRNA‐His‐GTG has a regulatory role in cellular senescence. Chai et al. found elevated levels of tiRNA‐His‐GTG‐001 expression in intestinal biopsy samples from patients with irritable bowel syndrome with diarrhea (Chai et al. 2021). KEGG enrichment analysis of its target genes revealed that they were enriched in the sphingolipid signaling pathway, MAPK signaling pathway, the tumor necrosis factor (TNF) signaling pathway, Retinoic acid‐inducible gene I (RIG‐I)‐like receptors signaling pathway, etc., and all of them have been proven to be important in regulating senescence (Anerillas et al. 2020; Gupta et al. 2003; Li et al. 2017; Molony et al. 2017; Trayssac et al. 2018). Distinct mechanisms in different disease scenarios may lead to discrepancies in the regulatory potential of 5′‐tiRNA‐His‐GTG in cellular senescence.

Protein expression analysis of photoaging cell models rescued with the 5′‐tiRNA‐His‐GTG Inhibitor revealed that NUP98 expression was elevated in this group (Figure 4E,H). At the post‐transcriptional level, tsRNAs can act in a manner similar to miRNAs by being sequence complementary to endogenous mRNA (Yang et al. 2023). tsRNAs affect mRNA stability by binding to the mRNA 3′ UTR region and thus participate in regulating gene expression. Bioinformatics tools identified that NUP98 may be one of the target genes of 5′‐tiRNA‐His‐GTG. The dual luciferase assay and the mRNA stability assay indicated direct binding of 5′‐tiRNA‐His‐GTG to the NUP98 mRNA 3′‐UTR and destabilization of NUP98 mRNA (Figure 4F,I,J). We found that NUP98 is one of the key target genes of 5′‐tiRNA‐His‐GTG.

NUP98 is a peripheral nuclear pore protein with a characteristic Gly‐Leu‐Phe‐Gly (GLFG) repeats structural domain. It mediates NPC selectivity and permeability and is involved in different cellular processes including nuclear import and export, mRNA export, mitotic progression, and gene expression regulation (Liu and Hetzer 2022). NUP98 expression level is reduced in senescent skin fibroblasts (Kim et al. 2010). We speculated that 5′‐tiRNA‐His‐GTG functions by decreasing the level of NUP98 mRNA expression and inhibiting its protein expression (Figure 4D,G). To further confirm the regulatory role of 5′‐tiRNA‐His‐GTG on NUP98, we conducted in vitro NUP98 rescue experiments. Overexpression of NUP98 could rescue HDF cells senescence induced by 5′‐tiRNA‐His‐GTG (Figure 5).

Colombo et al. identified two siblings with biallelic NUP98 germline variants. Both presented with sparse eyebrows and eyelashes, bilateral cataracts, and signs of premature aging. Protein molecular modeling studies of the mutated NUP98 variant revealed that the reduced intramolecular cohesion of the mutant protein leads to protein dysfunction and may impair protein–protein and protein‐RNA interactions (Colombo et al. 2023). We further found that overexpression of NUP98 attenuated the cellular senescence phenotype in both normal HDF cells and a UVB‐induced photoaging cell model (Figure 6 and Figure S3). The differentially expressed genes in HDF cells overexpressing NUP98 were enriched in cellular senescence, cell cycle, DNA replication, p53 signaling pathway, PI3K‐AKT signaling pathway, and MAPK signaling pathway (Figure 7B). A previous study found that knockdown of NUP98 in PC3 cells activated the JNK pathway (Pulianmackal et al. 2022). Our study revealed that HDF cells overexpressing NUP98 reduced the level of JNK signaling pathway activation (Figure 7C). In this study, the JNK signaling pathway was found activated in HDF cells after overexpression of 5′‐tiRNA‐His‐GTG, and such activation could be rescued by overexpressing NUP98 (Figure 7D,E). The JNK signaling pathway was also suppressed after transfecting 5′‐tiRNA‐His‐GTG Inhibitor in the photoaging cell model group (Figure 7G).

The JNK is a potential regulator in regulating senescence‐related processes, stressing the importance of JNK as a key target molecule in the study of senescence (Lee and Liu 2020). ROS generating, ECM degrading, and following elevated MMP expression are essential in the process of skin photoaging (Rittie and Fisher 2015). Accumulation of ROS inactivates protein tyrosine phosphatases (PTPs) which in turn activate receptor tyrosine kinases (RTKs). RTKs activate downstream Extracellular signal‐regulated kinase (ERK), p38, and JNK signaling pathways. The downstream transcription factor AP‐1 was activated to promote MMP‐1, 3, and 9 expression and inhibit procollagen‐1 expression (Toutfaire et al. 2017). JNK pathway activation also upregulates p53 to promote SASP factors expression (Zhang et al. 2020). Our study demonstrates that targeting NUP98 and JNK signaling pathways represents a key mechanism underlying 5′‐tiRNA‐His‐GTG‐induced cellular senescence.

There are also limitations in this study. In the photoaging cell model, multiple tRFs and tiRNAs expression differences were observed, and their biological functions need to be further explored. Research suggests that impaired protein synthesis affects cellular senescence, and 5′‐tiRNA triggers the phosphorylated eukaryotic translation initiation factor 2A (eIF2A) independent assembly of stress granules, inhibiting translation and thus overall protein synthesis (Ivanov et al. 2011). Whether 5′‐tiRNA‐His‐GTG affects amino acid translation and overall protein synthesis still requires more research. Although NUP98 was the interested gene in this study, it is necessary to conduct additional studies to investigate whether 5′‐tiRNA‐His‐GTG can interact with other mRNAs or proteins and thus contribute to its regulatory role.

## 4 Conclusion

To summarize, our study demonstrated that 5′‐tiRNA‐His‐GTG expression was elevated in photoaging cell models and the skin of photoaging animal models. 5′‐tiRNA‐His‐GTG induced cellular senescence by down‐regulating NUP98 expression and activating the JNK signaling pathway. Inhibition of 5′‐tiRNA‐His‐GTG ameliorates the UVB‐induced senescence phenotype in the photoaging cell model and UVB‐induced skin photoaging in nude mice. Targeting 5′‐tiRNA‐His‐GTG offers new therapeutic approaches for treating skin photoaging.

## 5 Materials and Methods

### 5.1 Cell Culture and UVB‐Induced HDF Photoaging Cell Model

Human dermal fibroblasts (HDF cells) were purchased (ZQXZBIO, Shanghai, China) and cultured as per the previous description (Chen et al. 2024). When the cell confluency reached 60%–70%, we removed the cell culture medium and replaced it with a thin layer of PBS. Subsequently, the HDF cells were exposed to UVB (30 mJ/cm^2^) with a cellular UVB irradiator (SIGMA High‐tech Co. Ltd., China). PBS was removed from the UVB‐radiated cells, and the complete medium was added, and the cells continued to be cultured in the same conditions for 24 h before the following experiments. The control group continued to be cultured under the same conditions.

### 5.2 Senescence‐Associated β‐Galactosidase (SA‐β‐gal) Assay

After the indicated treatment, SA‐β‐gal staining was performed to assess cellular senescence using a senescence‐β galactosidase staining kit (Beyotime, China) according to the manufacturer's protocol.

### 5.3 RNA Isolation and Quantitative Reverse Transcription‐PCR (qRT‐PCR) Assay

The total RNA of cells or skin samples was extracted by TRIzol (Invitrogen, USA). For tsRNAs and U6, Bulge‐loop qRT‐PCR Primer Sets specific (one RT primer and a pair of qPCR primers for each set) for tsRNAs and U6 were designed by RiboBio (Guangzhou, China) and ChamQ SYBR qPCR Master Mix (Without ROX) (Vazyme, China) was used. For mRNA, HiScript III All‐in‐one RT SuperMix Perfect for qPCR (Vazyme, China) and ChamQ SYBR qPCR Master Mix (Without ROX) were used. GAPDH/U6 was used as the reference gene for normalization. The primer sequences are provided in Table S1. Relative expression of mRNAs, tRNAs, and tsRNAs was calculated by the 2^(−ΔΔCt)^ method.

### 5.4 Western Blotting Assay

Cell and skin tissue protein samples were prepared, and western blotting assays were performed as previously described (Chen et al. 2024). Primary antibodies against p21 (10355‐1‐AP), p53 (10442‐1‐AP), and Collagen type I polyclonal antibody (14695‐1‐AP) were purchased from Proteintech (China). The primary antibody against NUP98 (EPR22818‐128) was purchased from Abcam (Cambridge, UK). Primary antibodies against SAPK/JNK (no. 9252S) and Phospho‐SAPK/JNK (Thr183/Tyr185) (no. 4668S) and anti‐rabbit IgG, HRP‐linked antibody (no. 7074) were purchased from Cell Signaling Technology (MA, USA). The ImageJ software quantifies the density of interested protein bands.

### 5.5 EdU Staining Assay

After the indicated treatments, cells were incubated with EdU (10 μM) for 2 h. The pre‐incubated cells were treated with the YF Click‐iT EdU Universal Cell Proliferation Detection Kit (C6044L; UElandy, China), based on the protocol. Images of the treated cells were acquired with a fluorescence microscope (Olympus, Japan).

### 5.6 tsRNAs High‐Throughput Sequencing

After the indicated treatments, total RNA samples of the UVB‐induced HDF photoaging model and the control group were isolated using TRIzol. Total RNA samples were pretreated with the following reagents to remove RNA modifications before library construction: 3′‐aminoacyl (charged) diacylation to 3′‐OH (hydroxyl group) for 3′ adaptor ligation; 3′‐cP (2′,3′‐cyclic phosphate) removal to 3′‐OH for 3′ adaptor ligation; 5′‐OH phosphorylation to 5′‐P for 5′‐adaptor ligation; and m1A and m3C demethylation for efficient reverse transcription. The completed sequencing libraries were constructed and quantified by Agilent 2100 Bioanalyzer. The completed small RNA sequencing was performed on the Illumina NextSeq 500 system (Illumina, CA, USA) conducted by Aksomics (Shanghai, China), with the NextSeq 500/550 V2 kit (no. FC‐404‐2005, Illumina, USA).

Raw data were examined by FastQC software and trimmed reads (pass Illumina quality filter, trimmed 5′, 3′ adaptor bases by cut adapt) were aligned to mature tRNA and precursor‐tRNA sequences in GtRNAdb and tRFMINTbase database with bowtie software. The abundance of tRFs and tiRNAs were evaluated and normalized as counts per million of total aligned reads (CPM). Finally, the Arraystar tRF & tiRNA‐seq data analysis package was employed for further data analysis. Differentially expressed tsRNAs (fold changes ≥ 1.5, *p* ≤ 0.05) were detected by the count value with R package edgeR. Principal Component Analysis (PCA), heatmap, Venn plots, Hierarchical clustering, and Volcano plots are performed in R or Perl environment for statistical computing and graphics of the expressed tsRNAs.

### 5.7 Animal Study

Eight 8‐week‐old female BALB/c nude mice were purchased from GemPharmatech Co. Ltd. (Jiangsu, China). Mice were allocated into UVB‐induced photoaging and control groups randomly after 1 week of resting. To induce skin photoaging, the dorsal skin of treated mice was irradiated with UVB 6 times a week for 5 consecutive weeks, with the cellular UVB irradiator described above. The initial radiation dosage was 120 mJ/cm^2^ for the first week, followed by weekly increases of 30 mJ/cm^2^. All mice were measured for transepidermal water loss levels (TWEL) in the dorsal skin using gpskin (GPOWER Inc., Republic of Korea) after completing the indicated treatments. All mice were sacrificed, and dorsal skin samples were collected for histological observations, as well as RNA and protein extraction. After paraffin embedding, skin tissues were subjected to H&E and Masson trichrome staining. The epidermal thickness was measured in five random fields to assess the epidermal thickening, and the collagen content was measured using ImageJ. We obtained approval for animal studies from the Animal Welfare Ethics Review Committee of the Institute of Dermatology, Chinese Academy of Medical Sciences (approval no. 2023‐KY‐057).

### 5.8 Adeno‐Associated Virus Transfection

Adeno‐associated virus (AAV) targeted to inhibit 5′‐tiRNA‐His‐GTG and negative control was purchased from Genechem Co. Ltd. (China). Eight 4‐week‐old female BALB/c nude mice were purchased from GemPharmatech Co. Ltd., and after 1 week of adaptive feeding, the dorsal skin of the mice received AAV transfection. 100 mL of AAV9‐5′‐tiRNA‐His‐GTG‐Inhibition‐EGFP (titer: 1 × 10^11^ vg/mL), AAV9‐control‐EGFP (titer: 1 × 10^11^ vg/mL), and NS were intradermally injected into the mice dorsal skin. After 3 weeks, the mice dorsal skin was obtained, and immunostaining of EGFP was performed to confirm the virus has been transfected in the dermis. Subsequently, the rest of the AAV‐treated mice continued with UVB radiation to induce skin photoaging as described above.

### 5.9 Cell Transfection and Lentiviral Transduction

HDF cells were transfected with 5′‐tiRNA‐His‐GTG mimic, Inhibitor, and their negative control (MimicNC, InhibitorNC) (GenPharma, China) with siRNA‐mate plus transfect reagent (GenPharma, China). The sequences of mimic and Inhibitor are listed in Table S2. To prepare lentivirus overexpressing NUP98 (oe‐NUP98), the NUP98 cDNA sequence, or NC sequence was cloned into the gcGFP‐IRES‐Puromycin lentivirus vector (Genechem, China) respectively. HDF cells were infected with recombinant lentivirus (MOI = 100) at 30%–40% confluence. Then, the infected cells were further cultured for selection using media containing 2 μL/mL puromycin.

### 5.10 Label‐Free Proteomics

After the indicated treatments, UHPLC–MS/MS analyses of cell protein samples of the UVB‐induced HDF photoaging model transfected with 5′‐tiRNA‐His‐GTG Inhibitor or InhibitorNC were performed using an EASY‐nLCTM 1200 UHPLC system (Thermo Fisher, Germany) coupled with an Orbitrap Exploris 480 mass spectrometer (ThermoFisher, Germany) in Novogene Co. Ltd. (Beijing, China). All the resulting spectra were searched against the UniProt database by the search engine: Proteome Discoverer2.5 (ThermoFisher, Germany). The proteins whose quantitation was significantly different between the HDF photoaging model transfected with the 5′‐tiRNA‐His‐GTG inhibitor or inhibitorNC groups (*p* < 0.05 and |log2FC| > 2.0, [foldchange, FC]) were defined as differentially expressed proteins (DEPs). DEPs were used for Volcanic map analysis and cluster heat map analysis.

### 5.11 RNA Stability Assay

In order to assay the stability of *NUP98* mRNA, HDF cells after indicated treatment were treated with 10 μL/ml Actinomycin D (HY‐17559, MCE) to block transcription. Cells were harvested after 0, 3 and 6 h incubation, and total RNA was isolated for RT‐qPCR (Ratnadiwakara and Anko 2018).

### 5.12 Reactive Oxygen Species Detection

After the indicated treatments, cells were incubated with Dihydroethidium (DHE, 10 μM) for 30 min (C1300‐2; APPLYGEN, China) according to the manufacturer's protocol. Images of the treated cells were acquired with a fluorescence microscope (Olympus, Japan).

### 5.13 Luciferase Reporter Assay

The predicted binding site of 5′‐tiRNA‐His‐GTG was constructed into the pmirGLO luciferase reporter vector (GenePharma, China). HEK293T cells were used for the luciferase assay. The pmirGLO luciferase vectors (pmirGLO‐NUP98 wild‐type or pmirGLO‐NUP98 mutant‐type) and 5′‐tiRNA‐His‐GTG Mimic or NC were co‐transfected into HEK293T cells. The Dual Luciferase Assay Kit (Promega, USA) was utilized to detect the luciferase activity.

### 5.14 RNA Sequencing and Analysis

After the indicated treatments, total RNA samples of oe‐NUP98 HDF cells and the control group were isolated using TRIzol. The completed small RNA was sequenced on the Illumina NextSeq 500 system (Illumina, CA, USA) at Tsingke Biotech, with the NextSeq 500/550 V2 kit (no. FC‐404‐2005, Illumina). Differentially expressed genes (DEGs) were defined as |log2FC| ≥ 1.5 and *Q* value ≤ 0.05. KEGG pathway enrichment analyses were performed.

### 5.15 Statistical Analysis

All experiments were conducted and replicated at least three times independently. The data are presented as the mean ± standard deviation (SD). In the Student's *t*‐test or one‐way ANOVA, *p* < 0.05 has been set as statistical significance. Significance levels were presented as follows: *p < 0.05, ** *p* < 0.01, and *** *p* < 0.001.

## Author Contributions

L.L. performed the majority of the experiments, analyzed data, and prepared the manuscript. Z.X., X.D., and L.C. isolated human dermal fibroblasts and conducted Western blot experiments. X.Z. performed a small part of qRT‐PCR. J.Z., C.L., and D.H. supervised the project. Y.H. and K.C. contributed to the study conception and supervision.

## Ethics Statement

This study was reviewed and approved by the Animal Welfare Ethics Review Committee of the Institute of Dermatology, Chinese Academy of Medical Sciences (approval no. 2023‐KY‐057).

## Conflicts of Interest

The authors declare no conflicts of interest.

## Supporting information


**Data S1.** Supporting Information.

## Data Availability

The data that support the findings of this study are available from the corresponding author upon reasonable request. The data are not publicly available due to privacy or ethical restrictions.

## References

[acel70049-bib-0001] Anerillas, C. , K. Abdelmohsen , and M. Gorospe . 2020. “Regulation of Senescence Traits by MAPKs.” Geroscience 42, no. 2: 397–408. 10.1007/s11357-020-00183-3. 32300964 PMC7205942

[acel70049-bib-0002] Beck, M. , and E. Hurt . 2017. “The Nuclear Pore Complex: Understanding Its Function Through Structural Insight.” Nature Reviews. Molecular Cell Biology 18, no. 2: 73–89. 10.1038/nrm.2016.147. 27999437

[acel70049-bib-0003] Chai, Y. , Y. Lu , L. Yang , et al. 2021. “Identification and Potential Functions of tRNA‐Derived Small RNAs (tsRNAs) in Irritable Bowel Syndrome With Diarrhea.” Pharmacological Research 173: 105881. 10.1016/j.phrs.2021.105881. 34509631

[acel70049-bib-0004] Chen, L. , Y. Hu , M. Zhang , et al. 2024. “METTL14 Affects UVB‐Induced Human Dermal Fibroblasts Photoaging via miR‐100‐3p Biogenesis in an m^6^A‐Dependent Manner.” Aging Cell 23, no. 5: e14123. 10.1111/acel.14123. 38380598 PMC11113260

[acel70049-bib-0005] Colombo, E. A. , M. Valiante , M. Uggeri , et al. 2023. “Germline NUP98 Variants in Two Siblings With a Rothmund‐Thomson‐Like Spectrum: Protein Functional Changes Predicted by Molecular Modeling.” International Journal of Molecular Sciences 24, no. 4: 4028. 10.3390/ijms24044028. 36835439 PMC9965077

[acel70049-bib-0006] Fitsiou, E. , T. Pulido , J. Campisi , F. Alimirah , and M. Demaria . 2021. “Cellular Senescence and the Senescence‐Associated Secretory Phenotype as Drivers of Skin Photoaging.” Journal of Investigative Dermatology 141, no. 4S: 1119–1126. 10.1016/j.jid.2020.09.031. 33349436

[acel70049-bib-0007] Gupta, S. , S. Chiplunkar , C. Kim , L. Yel , and S. Gollapudi . 2003. “Effect of Age on Molecular Signaling of TNF‐Alpha‐Induced Apoptosis in Human Lymphocytes.” Mechanisms of Ageing and Development 124, no. 4: 503–509. 10.1016/s0047-6374(03)00028-9. 12714259

[acel70049-bib-0008] Ha, S. G. , and S. V. Lee . 2023. “The Role of tRNA‐Derived Small RNAs in Aging.” BMB Reports 56, no. 2: 49–55. 10.5483/BMBRep.2022-0199. 36646437 PMC9978369

[acel70049-bib-0009] Hajialiasgary, N. A. , M. H. Soheilifar , and N. Masoudi‐Khoram . 2024. “Exosomes in Skin Photoaging: Biological Functions and Therapeutic Opportunity.” Cell Communication and Signaling: CCS 22, no. 1: 32. 10.1186/s12964-023-01451-3. 38217034 PMC10785444

[acel70049-bib-0010] Ivanov, P. , M. M. Emara , J. Villen , S. P. Gygi , and P. Anderson . 2011. “Angiogenin‐Induced tRNA Fragments Inhibit Translation Initiation.” Molecular Cell 43, no. 4: 613–623. 10.1016/j.molcel.2011.06.022. 21855800 PMC3160621

[acel70049-bib-0011] Ke, Y. , and X. J. Wang . 2021. “TGFbeta Signaling in Photoaging and UV‐Induced Skin Cancer.” Journal of Investigative Dermatology 141, no. 4S: 1104–1110. 10.1016/j.jid.2020.11.007. 33358021 PMC7987776

[acel70049-bib-0012] Kim, H. K. , J. H. Yeom , and M. A. Kay . 2020. “Transfer RNA‐Derived Small RNAs: Another Layer of Gene Regulation and Novel Targets for Disease Therapeutics.” Molecular Therapy 28, no. 11: 2340–2357. 10.1016/j.ymthe.2020.09.013. 32956625 PMC7647673

[acel70049-bib-0013] Kim, S. Y. , S. J. Ryu , H. J. Ahn , H. R. Choi , H. T. Kang , and S. C. Park . 2010. “Senescence‐Related Functional Nuclear Barrier by Down‐Regulation of Nucleo‐Cytoplasmic Trafficking Gene Expression.” Biochemical and Biophysical Research Communications 391, no. 1: 28–32. 10.1016/j.bbrc.2009.10.154. 19903462

[acel70049-bib-0014] Lee, L. , and S. Liu . 2020. “Pathogenesis of Photoaging in Human Dermal Fibroblasts.” International Journal of Dermatology and Venereology 3, no. 1: 37–42.

[acel70049-bib-0015] Lee, S. , J. Kim , P. N. Valdmanis , and H. K. Kim . 2023. “Emerging Roles of tRNA‐Derived Small RNAs in Cancer Biology.” Experimental & Molecular Medicine 55, no. 7: 1293–1304. 10.1038/s12276-023-01038-5. 37430089 PMC10393972

[acel70049-bib-0016] Li, D. , X. Gao , X. Ma , et al. 2024. “Aging‐Induced tRNAGlu‐Derived Fragment Impairs Glutamate Biosynthesis by Targeting Mitochondrial Translation‐Dependent Cristae Organization.” Cell Metabolism 36, no. 5: 1059–1075. 10.1016/j.cmet.2024.02.011. 38458203

[acel70049-bib-0017] Li, P. , Y. Gan , Y. Xu , et al. 2017. “The Inflammatory Cytokine TNF‐α Promotes the Premature Senescence of Rat Nucleus Pulposus Cells via the PI3K/Akt Signaling Pathway.” Scientific Reports 7, no. 1: 42938. 10.1038/srep42938. 28211497 PMC5314336

[acel70049-bib-0018] Liu, J. , and M. W. Hetzer . 2022. “Nuclear Pore Complex Maintenance and Implications for Age‐Related Diseases.” Trends in Cell Biology 32, no. 3: 216–227. 10.1016/j.tcb.2021.10.001. 34782239

[acel70049-bib-0019] Molony, R. D. , J. T. Nguyen , Y. Kong , R. R. Montgomery , A. C. Shaw , and A. Iwasaki . 2017. “Aging Impairs Both Primary and Secondary RIG‐I Signaling for Interferon Induction in Human Monocytes.” Science Signaling 10, no. 509: eaan2392. 10.1126/scisignal.aan2392. 29233916 PMC6429941

[acel70049-bib-0020] Pulianmackal, A. J. , K. Kanakousaki , K. Flegel , et al. 2022. “Misregulation of Nucleoporins 98 and 96 Leads to Defects in Protein Synthesis That Promote Hallmarks of Tumorigenesis.” Disease Models & Mechanisms 15, no. 3: dmm049234. 10.1242/dmm.049234. 35107131 PMC8938402

[acel70049-bib-0021] Ratnadiwakara, M. , and M. L. Anko . 2018. “mRNA Stability Assay Using Transcription Inhibition by Actinomycin D in Mouse Pluripotent Stem Cells.” Bio‐Protocol 8, no. 21: e3072. 10.21769/BioProtoc.3072. 34532533 PMC8342049

[acel70049-bib-0022] Rittie, L. , and G. J. Fisher . 2015. “Natural and Sun‐Induced Aging of Human Skin.” Cold Spring Harbor Perspectives in Medicine 5, no. 1: a015370. 10.1101/cshperspect.a015370. 25561721 PMC4292080

[acel70049-bib-0023] Russell‐Goldman, E. , and G. F. Murphy . 2020. “The Pathobiology of Skin Aging: New Insights Into an Old Dilemma.” American Journal of Pathology 190, no. 7: 1356–1369. 10.1016/j.ajpath.2020.03.007. 32246919 PMC7481755

[acel70049-bib-0024] Saikia, M. , R. Jobava , M. Parisien , et al. 2014. “Angiogenin‐Cleaved tRNA Halves Interact With Cytochrome c, Protecting Cells From Apoptosis During Osmotic Stress.” Molecular and Cellular Biology 34, no. 13: 2450–2463. 10.1128/MCB.00136-14. 24752898 PMC4054315

[acel70049-bib-0025] Soheilifar, M. H. , N. Masoudi‐Khoram , A. Shirkavand , and S. Ghorbanifar . 2022. “Non‐Coding RNAs in Photoaging‐Related Mechanisms: A New Paradigm in Skin Health.” Biogerontology 23, no. 3: 289–306. 10.1007/s10522-022-09966-x. 35587318

[acel70049-bib-0026] Toutfaire, M. , E. Bauwens , and F. Debacq‐Chainiaux . 2017. “The Impact of Cellular Senescence in Skin Ageing: A Notion of Mosaic and Therapeutic Strategies.” Biochemical Pharmacology 142: 1–12. 10.1016/j.bcp.2017.04.011. 28408343

[acel70049-bib-0027] Trayssac, M. , Y. A. Hannun , and L. M. Obeid . 2018. “Role of Sphingolipids in Senescence: Implication in Aging and Age‐Related Diseases.” Journal of Clinical Investigation 128, no. 7: 2702–2712. 10.1172/JCI97949. 30108193 PMC6025964

[acel70049-bib-0028] Yang, M. , Y. Mo , D. Ren , S. Liu , Z. Zeng , and W. Xiong . 2023. “Transfer RNA‐Derived Small RNAs in Tumor Microenvironment.” Molecular Cancer 22, no. 1: 32. 10.1186/s12943-023-01742-w. 36797764 PMC9933334

[acel70049-bib-0029] Zhang, L. , J. Liu , and Y. Hou . 2023. “Classification, Function, and Advances in tsRNA in Non‐Neoplastic Diseases.” Cell Death & Disease 14, no. 11: 748. 10.1038/s41419-023-06250-9. 37973899 PMC10654580

[acel70049-bib-0030] Zhang, Y. , S. Zhou , W. Cai , et al. 2020. “Hypoxia/Reoxygenation Activates the JNK Pathway and Accelerates Synovial Senescence.” Molecular Medicine Reports 22, no. 1: 265–276. 10.3892/mmr.2020.11102. 32377698 PMC7248463

