# Supplementary material for: Mechanistic Insights Into 5′‐tiRNA‐His‐GTG Mediated Activation of the JNK Pathway in Skin Photoaging
Source: Aging Cell. 2025 Mar 17;24(7):e70049. doi: 10.1111/acel.70049 (PMC12266759; doi:10.1111/acel.70049)
Supplement: Supplementary file 1 — Data S1. Supporting Information. [file ACEL-24-e70049-s001.docx]

TableS1 primers

| IL-1β Forward | TGATGGCTTATTACAGTGGCAA |
| --- | --- |
| IL-1β Reverse | TAGTGGTGGTCGGAGATTCG |
| IL-6 Forward | ACTCACCTCTTCAGAACGAATTG |
| IL-6 Reverse | CCATCTTTGGAAGGTTCAGGTTG |
| IL-8 Forward | ACTGAGAGTGATTGAGAGTGGAC |
| IL-8 Reverse | AACCCTCTGCACCCAGTTTTC |
| GAPDH Forward | AATCCCATCACCATCTTCCA |
| GAPDH Reverse | TGGACTCCACGACGTACTCA |
| NUP98 Forward | CCATCTATGGATGACCTTGCTAAA |
| NUP98 Reverse | TCCGACCAATAGTGAAATCAGAGA |
| tRNA-His-GTG Forward | GCCGTGATCGTATAGTGGTT |
| tRNA-His-GTG Reverse | GATTCGAACCGAGGTTGCT |

TableS2 The sequences of Mimic and Inhibitor

| 5’ - tiRNA-His-GTG Mimic | GCCGUGAUCGUAUAGUGGUUAGUACUCUGCGUUG |
| --- | --- |
| 5’ - tiRNA-His-GTG Mimic NC | UUGUACUACACAAAAGUACUG |
| 5’ - tiRNA-His-GTG Inhibitor | CAACGCAGAGUACUAACCACUAUACGAUCACGGC |
| 5’ - tiRNA-His-GTG Inhibitor NC | CAGUACUUUUGUGUAGUACAA |


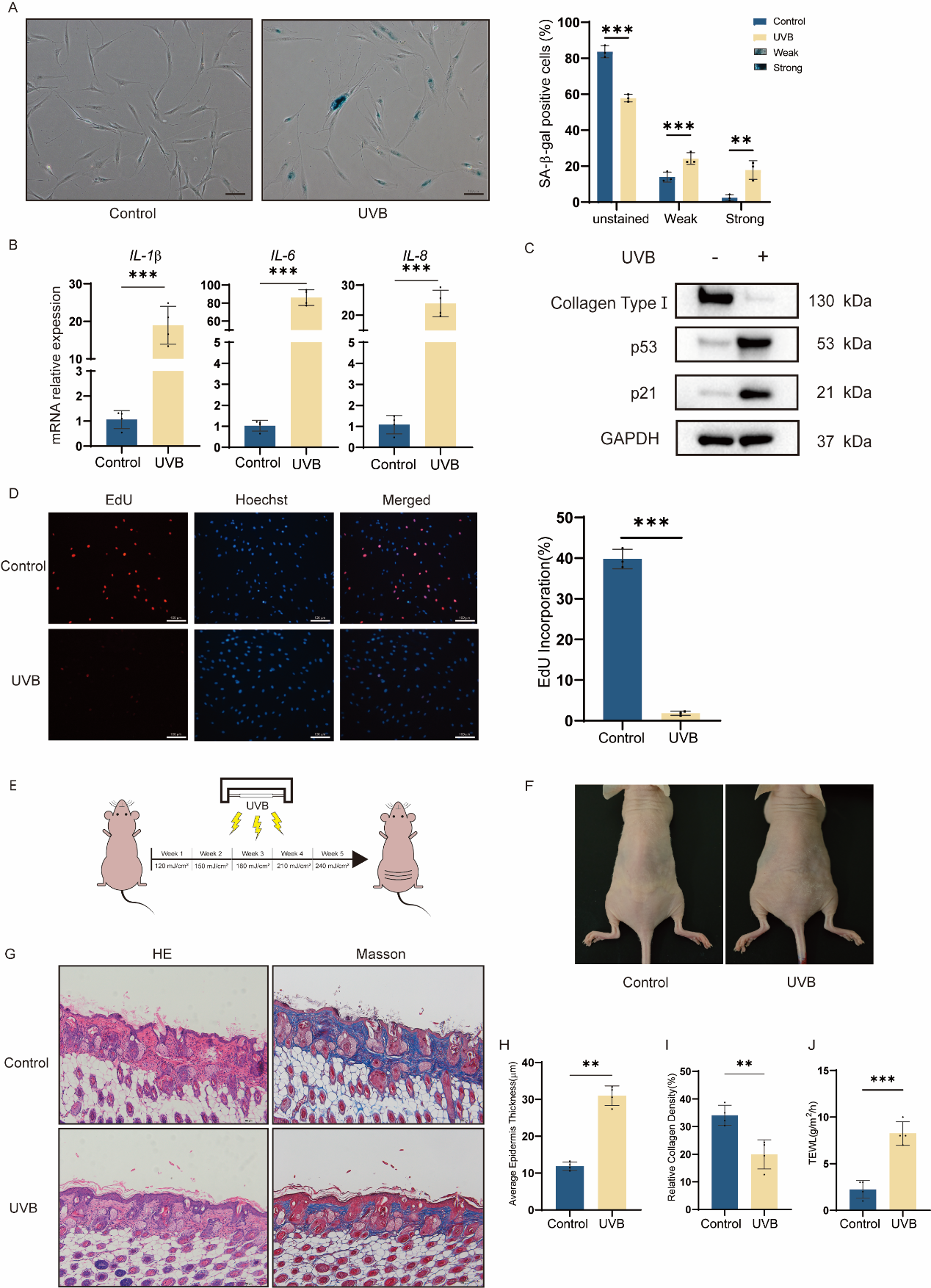


**Fig. S1 Construction of HDF cells and nude mice photoaging models using UVB radiation**

1. HDF cells were irradiated with UVB and stained with SA-β-gal after 24 h. Cells with blue staining represent senescent cells. The treat cells were divided into three groups, unstained, strongly positive, and weakly positive. The percentage of cells in each group was presented in a bar graph. The scale bar, 100μm.
2. The mRNA levels of *IL-1β*, *IL-6*, and *IL-8* mRNA in control group and photoaging cell model group.
3. Western blot analysis of Collagen Type I, p53, and p21 in HDF cells after UVB radiation.
4. Cell proliferation ability was analyzed using EdU assay. The scale bar, 100μm.
5. Schematic diagram of the protocol for using nude mice to construct an animal model of UVB-induced skin photoaging.
6. Representative images of dorsal skin of nude mice after UVB radiation.
7. Representative section images of mice dorsal skin, with H&E staining and Masson trichrome staining. The scale bar, 100μm.
8. Epidermal Thickness was measured in skin sections.
9. Collagen density in mice skin sections, measured by ImageJ.
10. The degree of transdermal water loss in the dorsal skin of mice was measured using the gpskin machine.


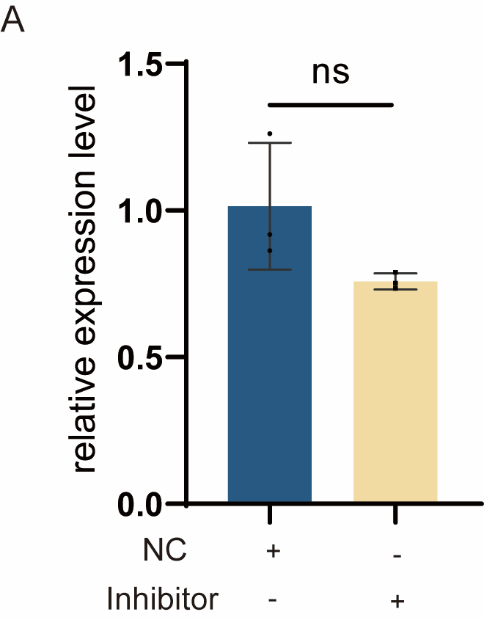


**Fig. S2 The expression levels of tRNA-His-GTG in various groups.**

1. The tRNA levels of tRNA-His-GTG in tiRNA Inhibitor group and NC group using qRT-PCR.


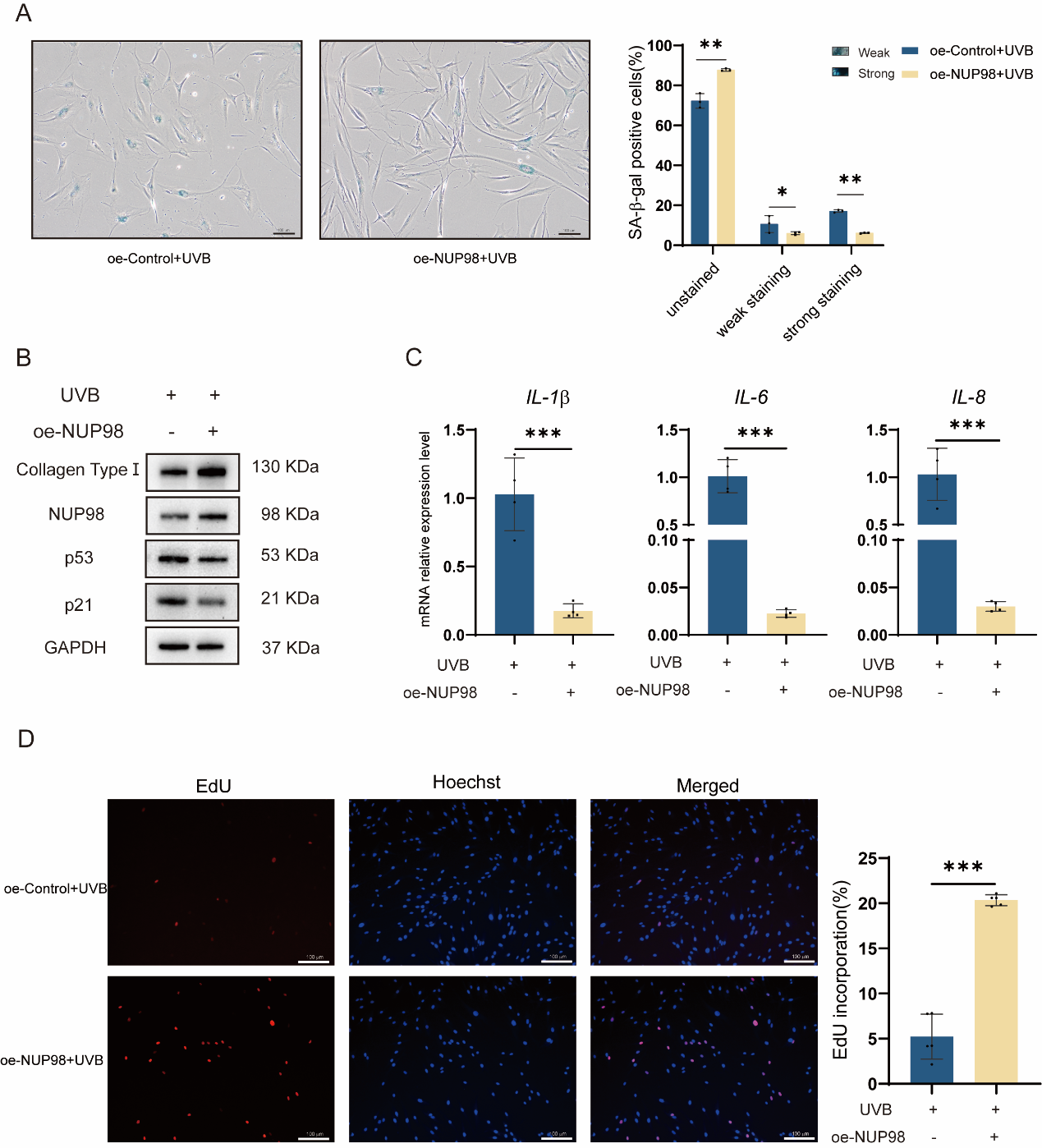


**Fig. S3 Overexpression of NUP98 attenuates UVB-induced HDF cellular senescence**

1. oe-NUP98 HDF cells radiated with UVB were stained with SA-β-gal after 48 h. Cells with blue staining represent senescent cells. The treated cells were divided into three groups, unstained, strongly positive, and weakly positive. The percentage of cells in each group was presented in a bar graph. The scale bar, 100 μm.
2. WB analysis of NUP98, Collagen Type I, p53, and p21 in oe-NUP98 HDF cells radiated with UVB.
3. The mRNA levels of *IL-1β*, *IL-6*, and *IL-8* mRNA in oe-NUP98 HDF cells radiated with UVB.
4. EdU assay was used to analyze oe-NUP98 HDF cells’ proliferation ability after UVB radiation. The scale bar, 100 μm.


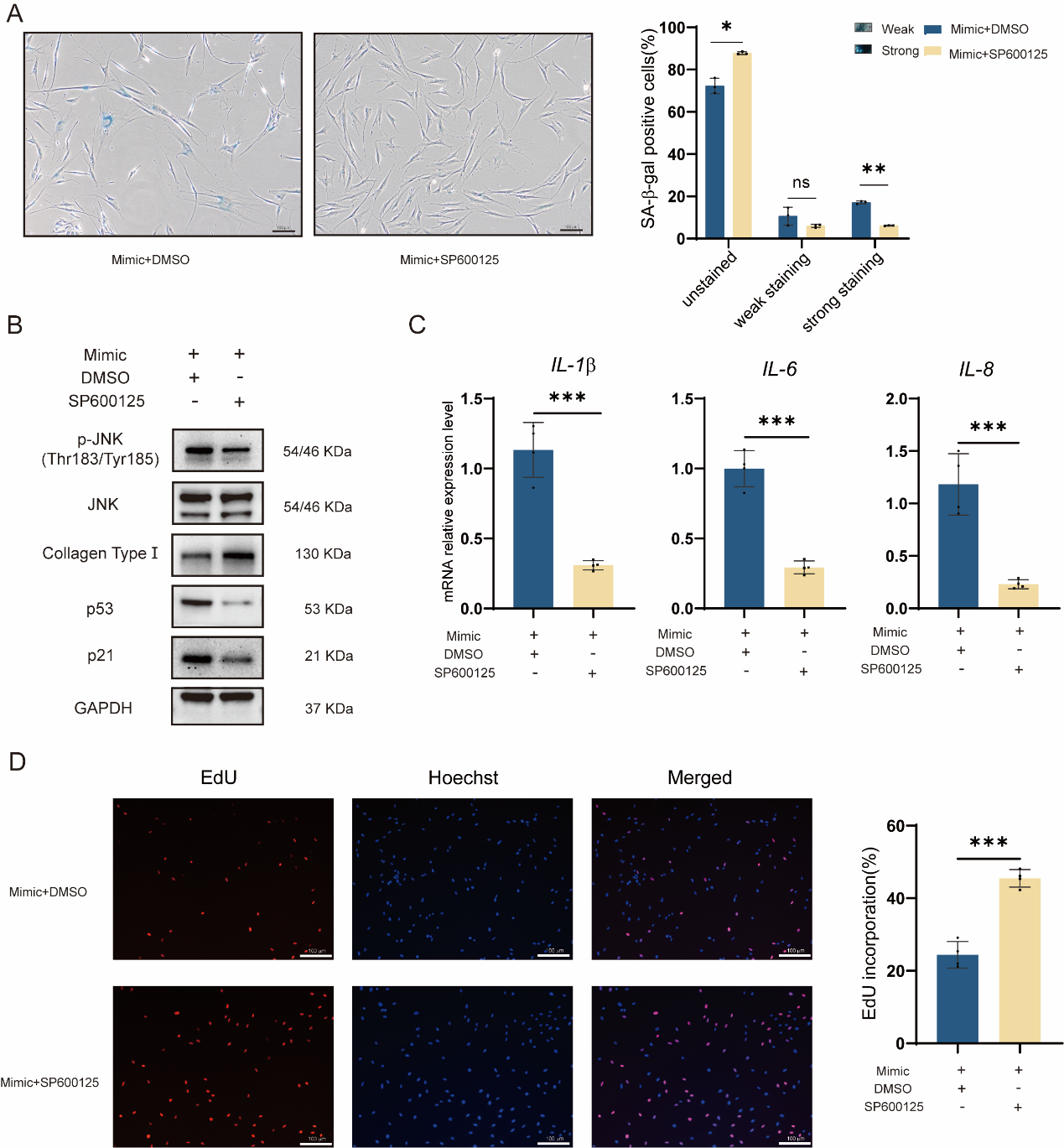


**Fig. S4 JNK signaling pathway inhibitor (SP600125) rescued 5’ - tiRNA-His-GTG-mimic-induced HDF cellular senescence**

1. Overexpressing 5’ - tiRNA-His-GTG HDF cells treated with JNK signaling pathway inhibitor (SP600125) were stained with SA-β-gal after 48 h. Cells with blue staining represent senescent cells. The treated cells were divided into three groups, unstained, strongly positive, and weakly positive. The percentage of cells in each group was presented in a bar graph. The scale bar, 100 μm.
2. WB analysis of JNK signaling pathway activation, Collagen Type I, p53, and p21 in overexpressing 5’ - tiRNA-His-GTG HDF cells treated with SP600125.
3. The mRNA levels of *IL-1β*, *IL-6*, and *IL-8* mRNA in in overexpressing 5’ - tiRNA-His-GTG HDF cells treated with SP600125.
4. EdU assay was used to analyze in overexpressing 5’ - tiRNA-His-GTG HDF cells’ proliferation ability after treating with SP600125. The scale bar, 100 μm.


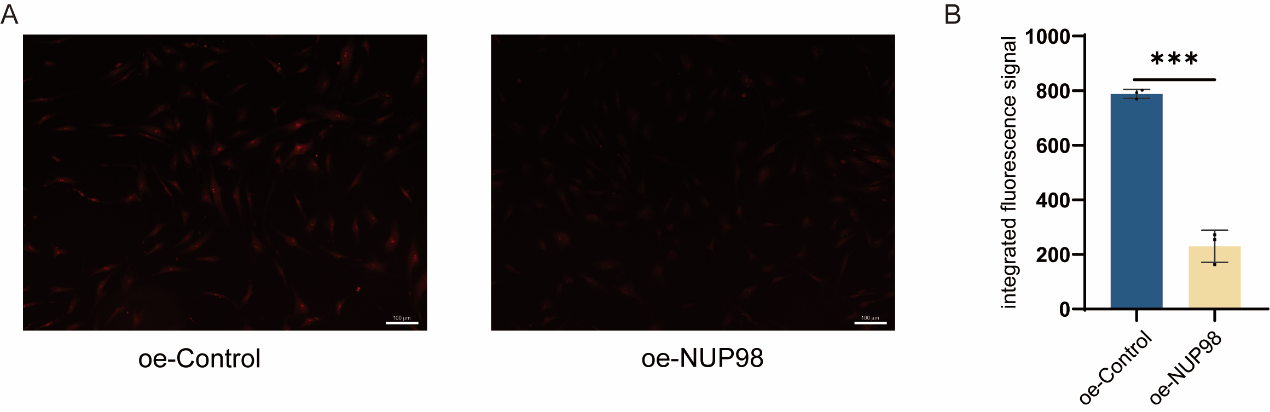


**Fig. S5 Overexpression of NUP98 attenuates ROS accumulation**

1. oe-NUP98 HDF cells incubated with Dihydroethidium (DHE, 10μM).
2. Fluorescence intensity of DHE in oe-NUP98 HDF cells was quantified by fluorescence microscope.


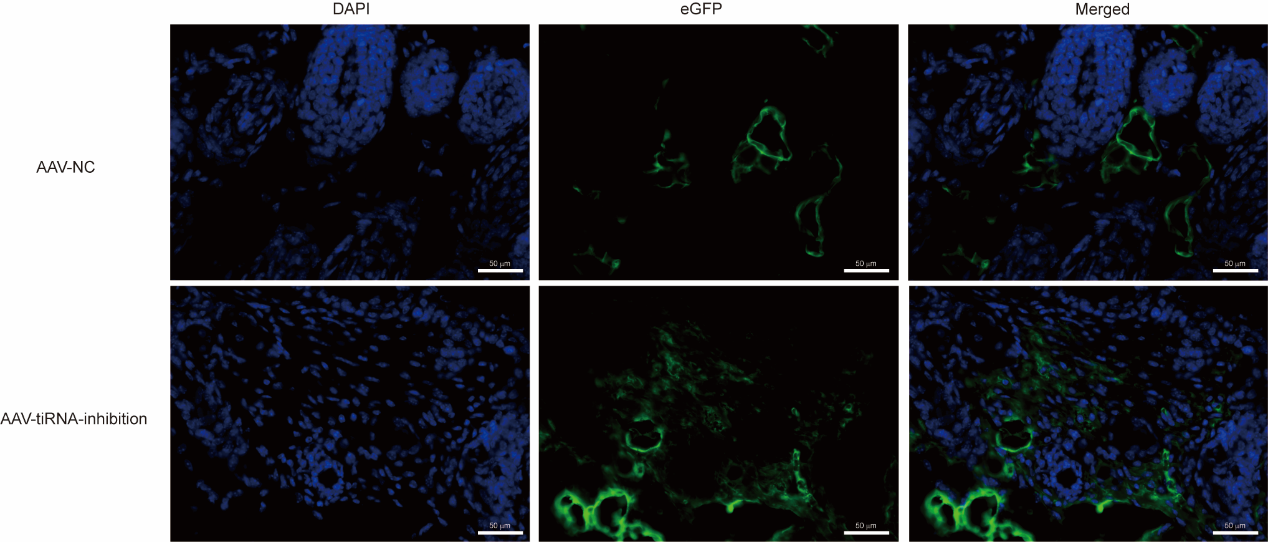


**Fig. S6 Dorsal skin of nude mice treated with AAV9-tiRNA-Inhibition**

The transfected mice skin tissues (AAV- AAV-5’ - tiRNA-His-GTG-Inhibition and AAV-NC) were observed under a fluorescence microscope); the green fluorescence is eGFP and the blue fluorescence is DAPI. The scale bar, 100 μm.
